# Transcriptional profiling and immunophenotyping show sustained activation of blood monocytes in subpatent *Plasmodium falciparum* infection

**DOI:** 10.1002/cti2.1144

**Published:** 2020-06-18

**Authors:** Jessica R Loughland, Tonia Woodberry, Matt Field, Dean W Andrew, Arya SheelaNair, Nicholas L Dooley, Kim A Piera, Fiona H Amante, Enny Kenangalem, Ric N Price, Christian R Engwerda, Nicholas M Anstey, James S McCarthy, Michelle J Boyle, Gabriela Minigo

**Affiliations:** ^1^ QIMR Berghofer Medical Research Institute Brisbane QLD Australia; ^2^ Menzies School of Health Research Darwin NT Australia; ^3^ Charles Darwin University Darwin NT Australia; ^4^ Australian Institute of Tropical Health and Medicine and Centre for Tropical Bioinformatics and Molecular Biology James Cook University Cairns QLD Australia; ^5^ John Curtin School of Medical Research Australian National University Canberra ACT Australia; ^6^ Timika Malaria Research Program Papuan Health and Community Development Foundation Timika Indonesia; ^7^ District Health Authority Timika Indonesia; ^8^ Centre for Tropical Medicine and Global Health Nuffield Department of Clinical Medicine University of Oxford Oxford UK; ^9^ Mahidol‐Oxford Tropical Medicine Research Unit Faculty of Tropical Medicine Mahidol University Bangkok Thailand; ^10^ College of Health and Human Sciences Charles Darwin University Darwin NT Australia; ^11^ Present address: The University of Newcastle Callaghan NSW Australia

**Keywords:** CHMI, interferon, malaria, monocytes, *Plasmodium falciparum*, RNA sequencing

## Abstract

**Objectives:**

Malaria, caused by *Plasmodium* infection, remains a major global health problem. Monocytes are integral to the immune response, yet their transcriptional and functional responses in primary *Plasmodium falciparum* infection and in clinical malaria are poorly understood.

**Methods:**

The transcriptional and functional profiles of monocytes were examined in controlled human malaria infection with *P. falciparum* blood stages and in children and adults with acute malaria. Monocyte gene expression and functional phenotypes were examined by RNA sequencing and flow cytometry at peak infection and compared to pre‐infection or at convalescence in acute malaria.

**Results:**

In subpatent primary infection, the monocyte transcriptional profile was dominated by an interferon (IFN) molecular signature. Pathways enriched included type I IFN signalling, innate immune response and cytokine‐mediated signalling. Monocytes increased TNF and IL‐12 production upon *in vitro* toll‐like receptor stimulation and increased IL‐10 production upon *in vitro* parasite restimulation. Longitudinal phenotypic analyses revealed sustained significant changes in the composition of monocytes following infection, with increased CD14^+^CD16^−^ and decreased CD14^−^CD16^+^ subsets. In acute malaria, monocyte CD64/FcγRI expression was significantly increased in children and adults, while HLA‐DR remained stable. Although children and adults showed a similar pattern of differentially expressed genes, the number and magnitude of gene expression change were greater in children.

**Conclusions:**

Monocyte activation during subpatent malaria is driven by an IFN molecular signature with robust activation of genes enriched in pathogen detection, phagocytosis, antimicrobial activity and antigen presentation. The greater magnitude of transcriptional changes in children with acute malaria suggests monocyte phenotypes may change with age or exposure.

## Graphical abstract

We investigate the transcriptional, functional and phenotypic changes in monocytes during an experimental blood‐stage *P. falciparum* infection in malaria naive healthy subjects and during an episode of clinical malaria in children and adults residing in a malaria‐endemic region. We specifically isolated monocytes for transcriptome analysis by RNA sequencing, to determine monocyte gene expression during subpatent and clinical malaria. Monocyte activation during subpatent malaria is driven by an inflammatory molecular signature and a greater magnitude of transcriptional changes in children with acute malaria suggest monocyte phenotypes may change with age or exposure.

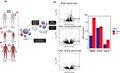

## Introduction

Malaria remains an important global disease, with an estimated 228 million cases and 405 000 deaths in 2018, the majority caused by *Plasmodium falciparum*.^1^ Upon transmission, *Plasmodium* parasites firstly infect hepatocytes to mature and multiply before release into the bloodstream and immediate infection of red blood cells (RBCs). Blood‐stage infection is characterised by cycles of asexual replication leading to RBC rupture and periodic malaria symptoms. Despite recent gains in reducing the burden of malaria, progress has stagnated in the last 3–5 years, with morbidity rising in several highly endemic countries.^1^ In areas of unstable malaria transmission, morbidity and mortality affect adults as well as children.

Innate and adaptive immune responses mediate both tolerogenic and antiparasitic protective mechanisms that can result in reduced clinical symptoms in future reinfections,^2^ yet acquisition of clinical immunity is complex and incompletely understood. Monocytes are integral to innate immune responses and may modulate adaptive immune responses during malaria.^3^, ^4^ An increased understanding of monocyte activation and function in subpatent *Plasmodium* infection may aid the development of strategies to enhance protective responses to improve the morbidity and mortality of malaria and assist in vaccine development.

Monocytes express a broad range of pattern recognition receptors, including toll‐like receptors (TLR),^5^ as well as receptors for the detection and phagocytosis of opsonised or non‐opsonised parasites or infected RBC.^6^, ^7^, ^8^ Besides parasite detection and phagocytosis, monocytes are major producers of predominantly inflammatory cytokines in malaria, including TNF and IL‐12; these assist immune control of *Plasmodium* infection,^9^, ^10^ but also contribute to the clinical symptoms of malaria.^11^, ^12^

Three distinct subsets of monocytes are identified by differential expression of the LPS receptor (CD14) and FcγRIII (CD16), and are defined as classical (CD14^+^CD16^−^), intermediate (CD14^+^CD16^+^) and non‐classical (CD14^−^CD16^+^) monocytes.^13^ These subsets have differing capacities to mediate innate and adaptive immune responses.^13^, ^14^ Classical monocytes mount a highly inflammatory response following exposure to bacterial TLR ligands,^14^ while non‐classical monocytes respond to viral antigens^15^ and intermediate monocytes are the most proficient at antibody‐mediated phagocytosis of *P. falciparum*.^8^, ^16^ *Plasmodium* infection can change the composition of circulating monocytes. In children with asymptomatic^17^ or uncomplicated malaria,^16^ and in malaria in pregnancy,^18^ CD16^+^ intermediate/non‐classical monocytes increase proportionally in the peripheral blood. However, there is a lack of information regarding prospective changes to monocyte composition during primary blood‐stage infection and acute malaria in non‐immune adults. Recent whole‐blood transcriptional analysis suggests that monocytes may have an anti‐inflammatory role in antimalarial immunity.^19^ Genetically susceptible Mossi groups in Mali lack transcriptionally active monocytes while, relatively resistant Fulani populations have transcriptionally active monocytes (but not T cells, B cells or NK cells) during *P. falciparum* infection^20^, highlighting the importance of monocytes in protective immune responses.

Here, we investigate the transcriptional, functional and phenotypic changes in monocytes during an experimental blood‐stage *P. falciparum* infection in malaria‐naive healthy subjects and during an episode of clinical malaria in children and adults residing in a malaria‐endemic region. We specifically isolated monocytes for transcriptome analysis by RNA sequencing, to determine monocyte gene expression during subpatent and clinical malaria. In primary infection, we focus on blood‐stage only infection, allowing us to examine how *P. falciparum* infection affects monocyte responses independent of the liver stage. Furthermore, our study design allows us to do paired analyses, as each patient's baseline or convalescence sample served as their own control.

## Results

### Monocyte gene expression during primary *P. falciparum* infection

**Figure 1:**
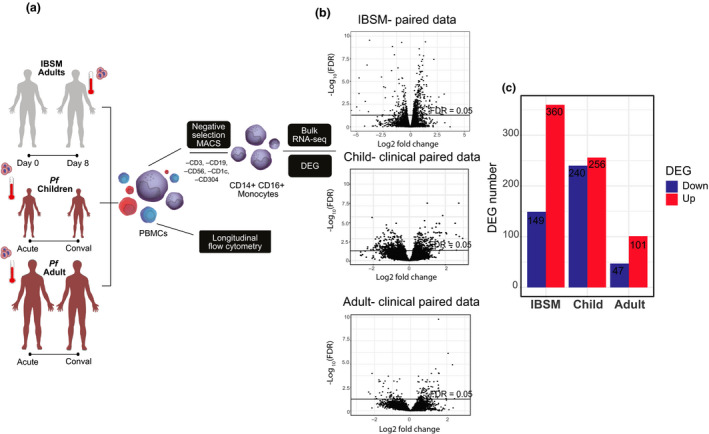
Cohort and RNA sequencing workflow. **(a)** Schematic of IBSM clinical trial cohort (grey) and acute clinical malaria trial cohorts with children (dark red) and adults (dark red). Monocytes were isolated by negative selection MACS separation and bulk‐RNAseq performed. Alongside, RNAseq monocyte number and activation were assessed by flow cytometry. **(b)** Total gene expression changes for IBSM, child and adult clinical cohorts, line indicates FDR < 0.05. **(c)** Total differentially expressed gene (DEG) numbers for the three cohorts, red indicates increased expression and blue indicates reduced expression. conval, convalescence (day 28); DEGs, differentially expressed genes; FC, fold change; FDR, false discovery rate; IBSM, induced blood‐stage malaria; PBMCs, peripheral blood mononuclear cells; Pf, *P. falciparum*.

To determine the immune pathways and genes activated early during a primary *P. falciparum* blood‐stage infection, we performed a paired transcriptome analysis of isolated monocytes via RNA sequencing. Blood monocytes were isolated from five volunteers at baseline immediately prior to intravenous infection with *P. falciparum* blood stages and on day 8 post‐infection allowing each individual's baseline sample to act as their own control (Figure 1a and b). On day 8 post‐infection, when the infection was still subpatent, 509 differentially expressed genes (DEGs, FDR < 0.05) were identified, of which 71% (360) were upregulated (Figure 1c, Supplementary table 1).

**Figure 2:**
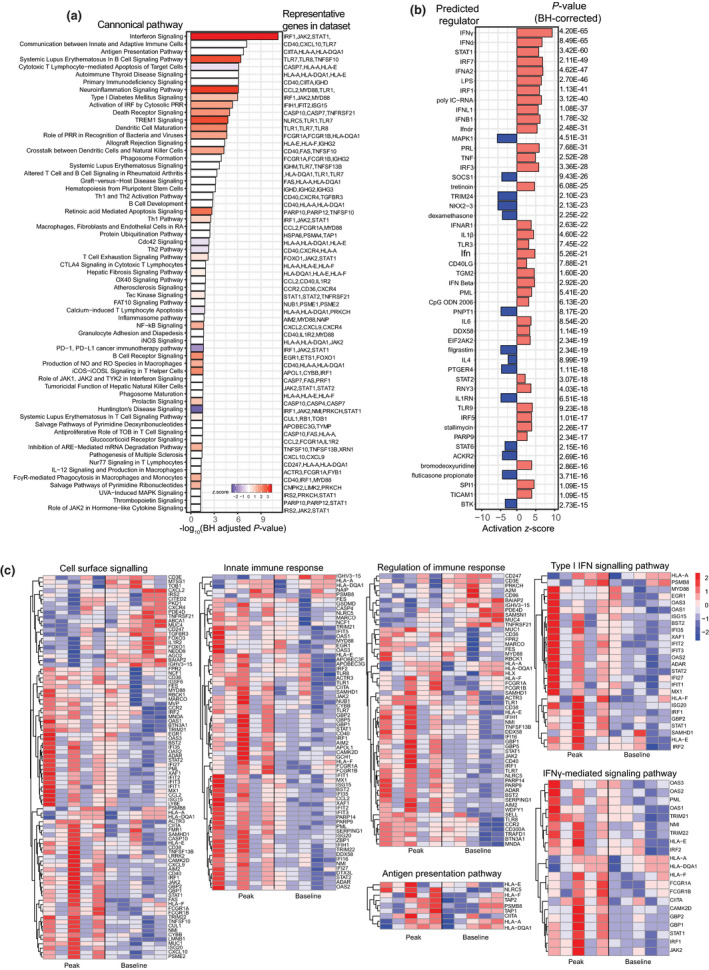
Transcriptional profile of monocytes after primary *P. falciparum* infection (IBSM cohort). **(a)** Canonical pathway analysis performed by IPA (Ingenuity Pathway Analysis), Benjamini–Hochberg‐adjusted significant pathways are shown, bar colour indicates activation *z*‐score (blue = reduced, white = 0 or no score and red = increased). **(b)** Predicted upstream regulators induced analysis performed using IPA, and red bars indicate activated, blue bars indicate inhibited, Benjamini–Hochberg‐adjusted *P*‐values. **(c)** Normalised gene expression of genes involved in enriched pathways represented in heat maps. Each column is one volunteer at ‘peak infection’ and ‘baseline’ (before infection). Red indicates increased gene expression, and blue indicates reduced gene expression. For all analyses, DEG input FDR < 0.01, *n* = 5 paired samples. DEGs, differentially expressed genes; FC, fold change; FDR, false discovery rate.

Using IPA (Ingenuity Pathway Analysis), we identified DEG enrichment of canonical pathways, including IFN signalling, communication between innate and adaptive immune cells (including *CD40*), antigen presentation, neuroinflammation signalling (including *CCL2*, *CD40*, *CXCR10*), triggering receptor expressed on myeloid cells‐1 (TREM‐1) signalling, role of pattern recognition receptors in recognition of bacteria and viruses (including *TLR1*, *TLR7*, *TLR8*), activation of IRF by cytosolic pattern recognition receptors, phagosome formation [including *FCGR1A* (CD64)], FcγR‐mediated phagocytosis and production of reactive oxygen (RO) species [including *CYBB*, the gene for NADPH oxidase 2 (NOX2) and *NCF1*, another subunit of NADPH oxidase] (Figure 2a and c). STRING analysis revealed similar GO (Gene Ontology) biological pathways relating to innate immune response (including *TLR1*, *TLR7*, *TLR8)*, cytokine‐mediated signalling, type I IFN signalling, regulation of immune system process, cell surface signalling (including *FCGR1A*, the gene for the high‐affinity Fc‐gamma receptor CD64; *CD36*, a receptor involved in non‐opsonised phagocytosis; and inflammatory chemotactic cytokines *CXCL9*, *CXCL10*), IFN‐γ‐mediated signalling, signal transduction, regulation of innate immune response [including *CD40* and signal transduction (including chemokine *CCL2* (MCP‐1) and its receptor *CCR2*)] (Figure 2c, Supplementary figure 1).

Using IPA, we further identified predicted upstream regulators of the DEGs (Figure 2b), with predicted upstream activation of IFN‐related genes (including *IFN‐α*, *IFN‐γ*, *IRF7*, *IFNA2*, *IRF1*, *IFNL1*, *IFNB1*, *IRF3*, *IFNαR*, *STAT1)*, and other inflammatory cytokines (*TNF*, *IL1B*, *IL6)*, pattern recognition receptors (*TLR3* and *TLR9)*, and TNF superfamily member *CD40LG* (CD40 ligand) (Figure 2b). Upstream regulators predicted as inhibited included mitogen‐activated protein kinase 1 (*MAPK1*), prostaglandin E2 receptor 4 (*PTGER4*), suppressor of cytokine signalling 1 (*SOCS1*) and interleukin 1 receptor antagonist (*IL1RN*), and tripartite‐motif protein 24 (*TRIM24*), the negative regulator of p53 stability.^21^ Increased TP53 along with increased p53 gene expression in monocytes has recently been identified to attenuate malaria‐induced inflammation.^19^

Across all identified pathways, the magnitude of response varied across participants (Figure 2c), consistent with the known heterogeneity of immune responses to primary *Plasmodium* infection in adults.^22^ However, overall, the monocyte transcriptional profile during subpatent *P. falciparum* blood‐stage infection is dominated by an IFN‐driven signature accompanied by pathways relating to pathogen detection, phagocytosis, antimicrobial activity and antigen presentation.

### Monocytes have an activated phenotype after a primary infection

**Figure 3:**
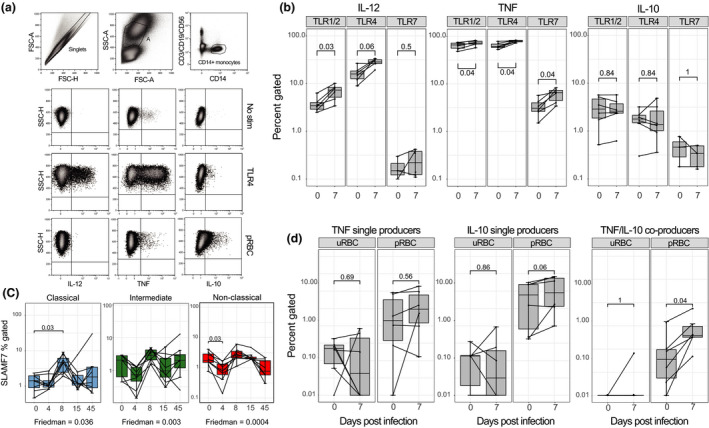
Monocytes increase cytokine production upon TLR stimulation during subpatent primary *P. falciparum* infection (IBSM cohort). **(a)** Representative gating of monocytes in whole‐blood intracellular cytokine assay. Monocytes were identified as lineage (CD3, CD19 and CD56)^−^, CD14^+^. Intracellular cytokine production by monocytes (IL‐12, TNF and IL‐10) in three conditions: no stimulation, TLR4 and pRBC stimulation. **(b)** Longitudinal monocyte responsiveness to TLR1/2 (Pam3CSK4/HKLM), TLR4 (LPS) or TLR7 (imiquimod) stimulation during IBSM, left plot; IL‐12 production, middle plot; TNF production, right plot; IL‐10 production. **(c)** Confirmation of SLAMF7 gene expression by flow cytometry on monocyte subsets: classical (blue), intermediate (green) and non‐classical (red), monocyte subset gating in Figure 4. The Friedman test was used to compare longitudinal data. **(d)** Monocyte cytokine production in response to uRBC or pRBC stimulation, left plot; TNF single producers, middle plot; IL‐10 single producers, right plot; TNF/IL‐10 co‐producers. Boxplot lower and upper hinges represent first and third quartiles with median line indicated across the box. Whisker lines correspond to highest and lowest values no further than 1.5 interquartile range from the hinges, whereas dot points beyond whisker lines are outliers. The Wilcoxon matched‐pairs sign‐rank test was used to compare paired data. Tests were two‐tailed and considered significant if *P*‐values < 0.05, *n* = 6 paired samples. FSC, forward scatter; pRBC, parasitised RBC; SSC, side scatter; TLR, toll‐like receptor; uRBC, uninfected RBC.

TLR1, TLR7 and TLR8 signalling pathways were enriched in monocytes during primary *P. falciparum* infection (Figure 2). To assess whether these transcriptional changes resulted in functional changes to TLR responsiveness, monocyte cytokine production was measured following stimulation with TLR agonists and *P. falciparum*‐infected RBCs (Figure 3a). TLR responsiveness was assessed on total CD14^+^ monocytes because surface expression of CD16 (FcγRIII) is rapidly lost during short‐term culture and stimulation.^23^ In response to TLR1/2 (PamCys2) or TLR4 (LPS) stimulation, monocytes produced a strong TNF and moderate IL‐12 response (Figure 3b), which increased at peak infection. In response to TLR7 (imiquimod) stimulation, monocytes had a modest TNF response, which significantly increased at peak infection (Figure 3b). TLR agonists induced low IL‐10 cytokine production which did not change following infection (Figure 3b). Increased expression of SLAMF7 on monocytes indicates activation specifically by TLR triggering.^24^ Consistent with increased *slamf7* gene expression (Figure 5c) and increased TLR responsiveness (Figure 3b), phenotypic expression of SLAMF7 was also increased at peak infection on classical monocytes (Figure 3c).

Monocyte responsiveness to pRBC *in vitro* stimulation at baseline and at peak infection was also assessed. Prior to infection, pRBC stimulation resulted in low but detectable parasite‐specific TNF and IL‐10 production [*P* = 0.06 and 0.03 for pRBC versus uninfected RBC (uRBC), respectively; Supplementary figure 2]. At peak infection, IL‐10 production increased (*P* = 0.06), with a significant increase in TNF/IL‐10 co‐production in response to pRBC restimulation (*P* = 0.04) (Figure 3d). There was no IL‐12 production detected in response to pRBC or uRBC stimulation (data not shown).

Taken together, these data suggest that during a subpatent *P. falciparum* blood‐stage infection, monocytes responded to TLR stimulation with increased inflammatory cytokine production, while they co‐produced regulatory cytokines in response to restimulation with *P. falciparum* parasites, that is increased IL‐10 producers, suggesting that during subpatent *P. falciparum* malaria monocytes develop a regulatory capacity, potentially to minimise tissue damage following chronic activation.^25^

### Phenotypic changes to monocyte subsets persist after parasite clearance

**Figure 4:**
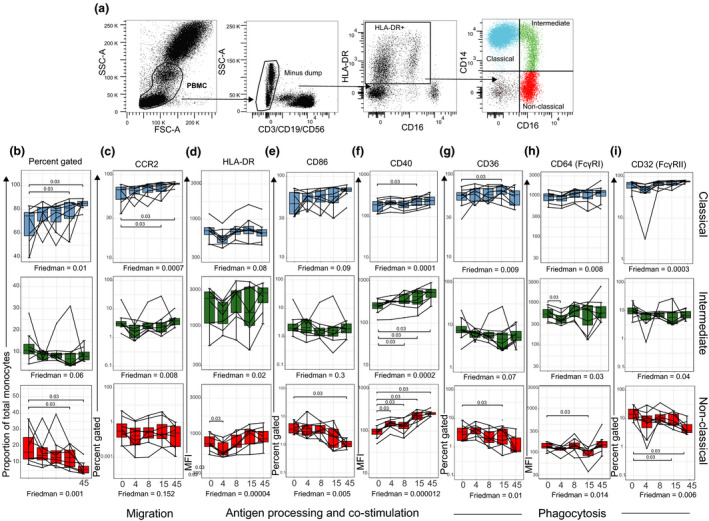
Differential activation of monocyte subsets during subpatent primary *P. falciparum* infection (IBSM cohort). **(a)** Fresh whole‐blood gating strategy for total monocyte subsets. Subsets were identified as lineage (CD3, CD19 and CD56)^−^, HLA‐DR^+^ and differential expression of CD14^+^ and CD16^+^. Classical (CD14^+^ CD16^−^), blue; intermediate (CD14^+^ CD16^+^), green; and non‐classical (CD14^dim^CD16^+^), red. **(b)** The proportion of monocyte subsets within the total monocyte population: classical (blue), intermediate (green) and non‐classical (red). Confirmation of genes differentially expressed in Figure 2 by flow cytometry, **(c)** CCR2 percent gated, **(d)** HLA‐DR MFI, **(e)** CD86 percent gated, **(f)** CD40 MFI, **(g)** CD36 percent gated, **(h)** CD64/FcyRI MFI and **(i)** CD32/FcyRII percent gated. Boxplot lower and upper hinges represent first and third quartiles with median line indicated across the box. Whisker lines correspond to highest and lowest values no further than 1.5 interquartile range from the hinges, whereas dot points beyond whisker lines are outliers. The Friedman test was used to compare longitudinal data. Tests were two‐tailed and considered significant if *P*‐values < 0.05, *n* = 8 paired samples. FSC, forward scatter; MFI, median fluorescence intensity; SSC, side scatter.

To determine whether the transcriptional changes we identified translated to phenotypic changes in monocytes, we assessed monocyte subsets (Figure 4a) for cell surface marker expression, prior to, during and after infection by flow cytometry. Despite monocyte subset frequency normally being tightly regulated,^14^ during a primary subpatent *P. falciparum* infection, classical (CD14^+^CD16^−^) monocyte frequency increased significantly on day 15 and remained significantly elevated at 45 days after infection (Figure 4b), whereas non‐classical (CD14^dim^CD16^+^) monocytes showed a corresponding decrease (Figure 4b). This increase in classical monocytes was also observed when classical monocytes were enumerated to all live PBMCs (Supplementary figure 3).

We further measured cell surface marker expression relating to activated pathways. CC‐chemokine receptor 2 (*CCR2*) gene expression was significantly increased in isolated monocytes at day 8 of blood‐stage infection. In accordance with the literature,^26^, ^27^ we observed CCR2 cell surface expression predominantly on classical monocytes, with a significant increase from day 15, which remained elevated at day 45 (Figure 4c). In line with the increased *CD40* gene expression following *P. falciparum* infection, we observed increased CD40 cell surface expression on all three monocyte subsets, with expression also remaining significantly elevated 45 days after infection (Figure 4f). This increase was especially prominent on intermediate (CD14^+^CD16^+^) and non‐classical (CD14^dim^CD16^+^) monocytes. CD40 is a member of the TNF receptor family, and increased expression of CD40 on monocytes is reported to increase monocyte pro‐inflammatory cytokine production and expression of other co‐stimulatory receptors.^28^ The increase in CD40 was not accompanied by an increase in co‐stimulatory molecule CD86 or HLA‐DR expression (Figure 4d and e).

We also assessed expression of CD36 which mediates non‐opsonic phagocytosis of parasites^29^ and CD64/FcγRI and CD32/FcγRII which are involved in opsonic phagocytosis.^8^ CD36 was differentially expressed across monocyte subsets, with classical monocytes expressing high levels of CD36, followed by intermediate monocytes, while < 10% of non‐classical monocytes expressed CD36 (Figure 4g). On classical monocytes, CD36 expression increased further by day 15 post‐infection. Classical and intermediate monocytes predominantly expressed CD64/FcγRI (Figure 4h). CD32/FcγRII expression was increased on classical monocytes but decreased on non‐classical monocytes, with significant changes observed at day 45 post‐infection (Figure 4i).

Taken together, these data indicate that the transcriptional changes observed during subpatent primary *P. falciparum* infection (Figures 1 and 2) result in multiple sustained changes to the composition and phenotypes of monocytes, suggestive of changes to innate immune effector mechanisms, antigen capture and cell migration pathways which are maintained after parasite clearance.

### Monocyte gene expression following naturally acquired acute uncomplicated malaria

Following the investigation of monocyte responses during a first *P. falciparum* blood‐stage infection in previously malaria‐naive adult volunteers, we next isolated monocytes from ten adults and eight children living in malaria‐endemic Papua who presented to the clinic with acute *P. falciparum* malaria and again 28 days after malaria treatment and successful parasite clearance (Figure 1a, Table 1). Lowland Papua is an area of perennial, unstable malaria transmission, where both children and adults are susceptible to symptomatic malaria because of incomplete development of immunity.

**Table 1:** Characteristics of participants

	Malaria‐naive volunteers (IBSM)	Malaria‐exposed adults (acute malaria)	Malaria‐exposed children (acute malaria)	*P*‐value^a^
Number	19	15	8	
Median age in years [IQR]	23 [20–26]	30 [20–38]	11 [8–13]	
Male, number (%)	17 (89)	10 (66)	3 (38)	
Median parasite density^b^ (parasites per μL) [IQR]	10 [4–19]	2275 [788–5712]	7377 [2965–13 644]	0.03

IBSM, induced blood‐stage malaria; IQR, interquartile range.

^a^ Mann–Whitney *U*‐test compared malaria‐exposed adults and malaria‐exposed children at presentation to clinic.

^b^ Determined by PCR for malaria‐naive adults (IBSM) and by microscopy for all other groups.

**Figure 5:**
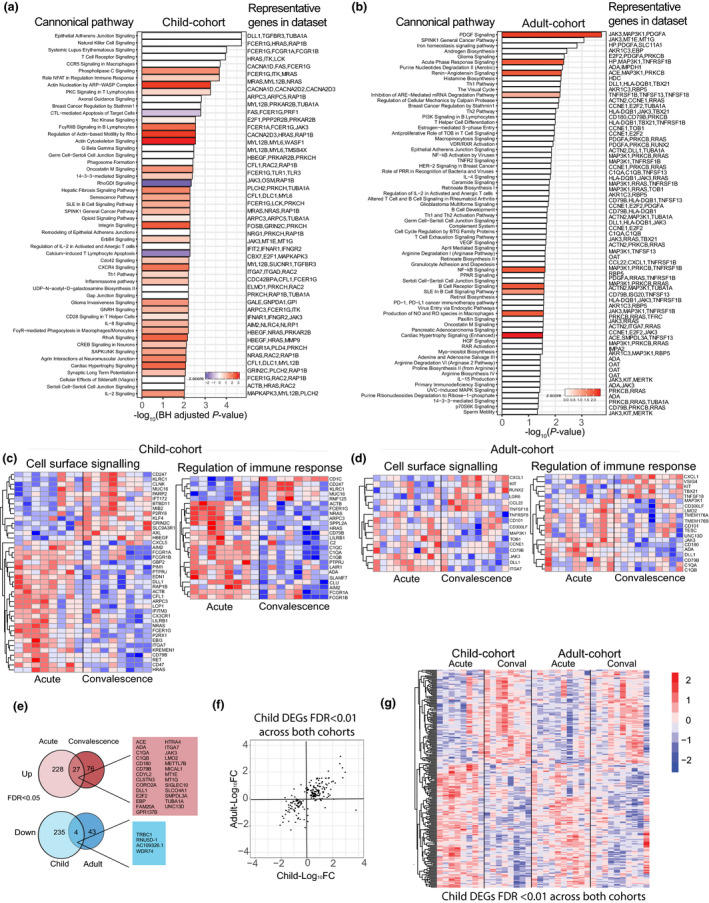
Transcriptional profile of monocytes in children and adults with acute clinical malaria (clinical malaria cohort). **(a)** Canonical pathway analysis performed by IPA, Benjamini–Hochberg‐adjusted significant pathways are shown, and bar colour indicates activation *z*‐score (blue = reduced, white = 0 or no score and red = increased). **(b)** Canonical pathway analysis performed by IPA, Fisher's exact test, significant pathways are shown, and bar colour indicates activation *z*‐score (blue = reduced, white = 0 or no score and red = increased). Normalised gene expression of genes involved in enriched pathways represented in heat maps. Each column is one patient at ‘acute’ infection and ‘convalescence’, 28 days after treatment. Red indicates increased gene expression, and blue indicates reduced gene expression. **(c)** Child cohort and **(d)** adult cohort. **(e)** Venn diagrams of DEGs in adult and child cohorts, red indicates increased gene expression and blue indicates reduced gene expression, FDR < 0.05. **(f)** Scatter plot showing fold change in DEGs across both adult and child cohorts, FDR < 0.01. **(g)** Heat map of normalised gene expression across child and adult acute malaria cohorts, FDR < 0.01, child cohort; *n* = 8 paired samples, adult cohort; *n* = 10 paired samples. conval, convalescence (day 28); DEGs, differentially expressed genes; FC, fold change; FDR, false discovery rate; Pf, *P. falciparum*.

We performed a paired analysis of transcriptional changes in isolated monocytes via RNA sequencing allowing each patient's convalescence sample to act as their own control. Overall, children had more differentially expressed genes at acute infection than adults (children = 496, adults = 148, FDR < 0.05, Figure 1b and c, Supplementary tables 2 and 3). In children with acute malaria, more IPA canonical pathways were predicted to be upregulated, including actin‐related pathways, integrin, CXCR4, IL‐8 signalling and Fcγ receptor‐mediated phagocytosis in macrophages and monocytes (Figure 5a), reflective of the larger number of DEGs identified. As seen in subpatent primary *P. falciparum* infection (Figure 2b), IPA‐predicted activated upstream regulators included IFN‐γ‐related gene products, including *IFNG* and *STAT1* in children (Supplementary figure 4b); in adults, IL‐4, IL‐6, the corresponding signal transducers STAT6 and STAT3, and triggering receptor expressed on myeloid cells‐1 (TREM1) (Supplementary figure 5b), a pathway activated in subpatent primary infection (Figure 2a). In adults with acute malaria, IPA canonical pathway analysis identified upregulation of pathways related to platelet‐derived growth factor (PDGF) signalling, acute‐phase response signalling [including *HP*, the gene for haptoglobin; *TNFRSF1B*, the gene for TNF receptor 2 (TNFR2); and mitogen‐activated protein kinase kinase kinase 1 (*MAP3K1*), a molecule involved in TNFR signalling] and NF‐κB signalling [including *MAP3K1*, *TNFRSF1B* and *PRKCB* (protein kinase C beta type)]. *OAT*, the gene for ornithine aminotransferase, an enzyme involved in L‐proline metabolism from L‐ornithine, was also differentially expressed in adults, suggestive of arginine degradation (Figure 5c).

As seen in experimental infection, STRING analysis showed DEGs were enriched for immune response pathways and more specifically, pathways involved in leucocyte activation [including Notch receptor ligand delta‐like 1 (*DLL1)* in both adults and children, and *SLAMF7* and *FCER1G*, the Fc receptor gamma chain (FcRγ)^30^ in children], regulation of immune response, complement activation and cell surface receptor signalling [including *FCGR1A*, the gene for the high‐affinity Fc‐gamma receptor CD64, and *FCGR1B* (CD64b) in children] (Supplementary figures 4 and 5c,d). More pathways were enriched in children when compared to adults (Figure 5a and c, Supplementary figures 4a and 5a). Of the DEGs, only 27 upregulated DEGs were shared between children and adults (FDR < 0.05, Figure 5e), including *C1QA* and *C1QB* encoding C1q, the first component of the classical complement pathway and *DLL1*.

Overall, despite not sharing many significantly deferentially expressed genes at FDR < 0.05, of the genes significantly differentially expressed in children with acute malaria, the direction of the fold change of each individual gene was positively correlated between children and adults (Figure 5f and g), suggesting that the pattern of gene changes is independent of age, but the magnitude of changes may be reduced in adults (Figure 5g).

### Intermediate monocytes increased in children with clinical malaria

**Figure 6:**
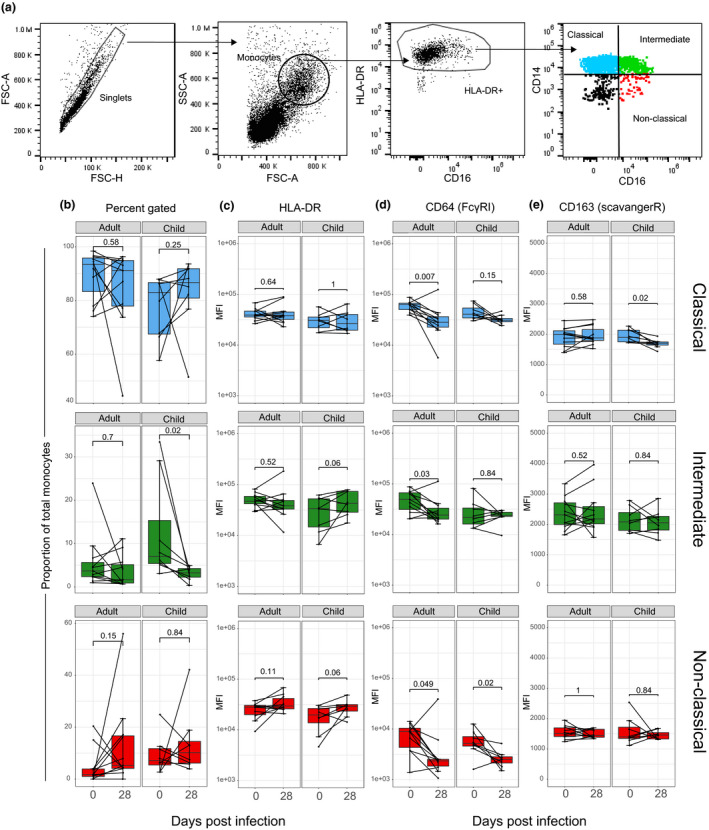
Increased intermediate monocytes in children with acute clinical malaria (clinical malaria cohort). **(a)** PBMC gating strategy for blood monocyte subsets. Monocyte subsets were identified by differential expression of CD14^+^ and CD16^+^. Classical (CD14^+^ CD16^−^), blue; intermediate (CD14^+^ CD16^+^), green; and non‐classical (CD14^dim^CD16^+^), red. **(b)** The proportion of monocyte subsets within the total monocyte population: classical (blue), intermediate (green) and non‐classical (red). Surface expression of **(c)** HLA‐DR MFI, **(d)** CD64/FcyRI MFI and **(e)** CD163 (scavenger receptor) MFI. Boxplot lower and upper hinges represent first and third quartiles with median line indicated across the box. Whisker lines correspond to highest and lowest values no further than 1.5 interquartile range from the hinges, whereas dot points beyond whisker lines are outliers. The Wilcoxon signed‐rank test was used to compare longitudinal data. Tests were two‐tailed and considered significant if *P*‐values < 0.05, child cohort; *n* = 8 paired, adult cohort; *n* = 11 paired samples. FSC, forward scatter; MFI, median fluorescence intensity; SSC, side scatter.

In addition to transcriptional changes, we also assessed monocyte subset phenotype in children and adults with malaria and at convalescence (Figure 6a). The proportion of intermediate (CD14^+^CD16^+^) monocytes was significantly higher in children during acute infection than convalescence (*P* = 0.02; Figure 6b), while we observed no significant changes in monocyte subsets in adult patients. The increase in intermediate monocytes in children during clinical infection is consistent with previous findings in uncomplicated malaria in Kenyan children,^16^ during asymptomatic infection,^17^ and in pregnant woman.^18^ There were no statistically significant changes in markers associated with antigen presentation (HLA‐DR, Figure 6c) or co‐stimulatory capacity (CD86, Supplementary figure 6). However, consistent with transcriptional upregulation of CD64/FcγRI genes *FCGR1A* and *FCER1G* (common FcRγ chain) in children (Figure 5b), CD64/FcγRI cell surface expression on monocyte subsets was increased in both children and adults during malaria (Figure 6d). Expression of the haemoglobin/haptoglobin scavenger receptor CD163 on classical monocytes was significantly increased in children, but not adults with acute malaria (Figure 6e). While the CXCR4 signalling pathway was upregulated in children with acute malaria (Supplementary figure 4a), we saw no significant change in CXCR4 expression on the cell surface (mobilises monocytes to bone marrow) across any of the monocyte subsets during acute malaria infection (Supplementary figure 6). One predicted activated upstream regulator in adults was IL‐4 (Supplementary figure 5b). IL‐4 can polarise monocytes towards alternative activation and was recently associated with nitric oxide insufficiency and disease severity.^31^ However, no significant change in monocyte surface expression of the mannose receptor CD206, a marker of alternatively activated monocytes,^32^ was observed in children or adults with uncomplicated acute malaria (Supplementary figure 6c).

## Discussion

Here, we evaluated transcriptional, phenotypic and functional monocyte responses in previously malaria‐naive volunteers undergoing their first *P. falciparum* blood‐stage infection. Our findings show that overall monocytes are transcriptionally, functionally and phenotypically activated during subpatent *P. falciparum* infection independent of *Plasmodium* liver stages. We identified robust activation of genes enriched in pathogen detection, phagocytosis, antimicrobial activity and antigen presentation. During primary infection, monocytes displayed heightened responsiveness to TLR stimulation and increased IL‐10 production upon re‐exposure to *P. falciparum*‐infected RBCs. Transcriptional activation resulted in compositional and phenotypical changes to monocyte subsets, suggesting altered migration, antigen presentation and phagocytosis function. In clinical malaria, the monocyte transcriptional response to infection appeared independent of age; despite more DEGs in children, the overall direction of transcriptional change was similar between adults and children. Together, these data provide an extensive map as to how human monocytes respond to *P. falciparum* infection and indicate monocytes have multiple roles in *Plasmodium* infection.

The monocyte response during subpatent primary *P. falciparum* was distinctively driven by a strong IFN molecular signature, including type I IFNs and gamma IFN, with the most activated pathways being interferon signalling and TREM1 signalling. The same two pathways were the most activated in a whole‐blood RNA sequencing analysis of healthy volunteers experiencing symptomatic malaria following controlled *P. falciparum* sporozoite infection.^33^ This strong alignment with our findings in isolated monocytes suggests blood monocytes are the key responders during blood‐stage malaria despite their relatively small abundance amongst circulating white blood cells. Furthermore, we now report this response as activated at subpatent parasitaemia, independent of *Plasmodium* liver stages. We observed a strong inflammatory gene signature in isolated monocytes during subpatent infection, including increased CXCL10, CXCL9 and STAT1 gene expression. In contrast, the strong transcriptional response in monocytes we report here is absent in plasmacytoid dendritic cells, which have minimal gene expression changes within the same study cohorts.^34^ These data extend previous research detailing PBMC or whole‐blood gene expression profiles in malaria patients^33^, ^35^, ^36^, ^37^, ^38^ and are consistent with existing knowledge on the importance of monocytes in inflammatory responses during malaria.^19^, ^20^

In line with IFN‐γ having the highest activation score of the predicted upstream regulators, monocytes displayed a heightened responsiveness to TLR ligands during subpatent infection. This is in accordance with previous reports of PBMC hyper‐responsiveness to TLR ligation in febrile malaria.^39^, ^40^ In contrast, monocyte responsiveness to restimulation with parasite‐infected RBC is skewed towards a regulatory phenotype with increased IL‐10 production, similar to our previous reports for CD16^+^ dendritic cells (DCs).^23^ The blunted inflammatory cytokine response to parasite restimulation may be explained by the enrichment of type I IFN signalling pathways, as IL‐6 production by monocytes is enhanced when type I IFN receptor is blocked.^41^ During malaria, type I IFN is produced by multiple cell types including T cells, NK cells and monocytes.^41^ While we did not evaluate monocyte cytokine production in children with uncomplicated clinical malaria, previous studies have demonstrated that monocytes isolated from children with uncomplicated malaria show increased IL‐12, IL‐6 and TNF cytokine production in response to TLR1/2 or TLR4 stimulation.^16^ In contrast, a separate study showed that monocytes from children with severe malaria had an impaired ability to produce inflammatory cytokines (TNF and IL‐6) to *in vitro* TLR agonists such as LPS.^10^ These differences in TLR responsiveness suggest that TLR signalling is influenced by parasite density and/or parasite exposure.

Besides IFNs, the CD40/CD40 ligand axis may contribute to monocyte activation following *Plasmodium* infection, with *CD40LG* being amongst the predicted activated upstream regulators. CD40 is considered a marker of monocyte activation, and CD40 ligation increases monocyte production of inflammatory cytokines^28^ and prostaglandin E2.^42^ CD40 may further contribute to monocyte interaction with activated endothelial cell and assist extravasation.^43^ We demonstrated upregulation of its ligand CD40 at the protein level on all three monocyte subsets, with the greatest increase seen on non‐classical monocytes, a monocyte subset that proportionally declined in the circulation over time. Whether this decline is related to extravasation or sequestration remains to be determined.

Predicted inhibited upstream regulators of monocyte gene expression in subpatent primary infection included MAPK1, IL1RN and TRIM24, which overlapped with regulators predicted to be inhibited in malaria‐naive volunteers experiencing symptomatic malaria following *P. falciparum* sporozoite infection.^33^ TRIM24 targets p53 degradation,^21^ a molecule recently reported to play a role in clinical immunity, as p53 is upregulated at RNA and protein level in asymptomatic *P. falciparum* carriers during the malaria season in Mali.^19^ The congruent overlap of upstream regulators between these studies suggests these predicted inhibited upstream regulators take effect early during blood‐stage infection, independent of *P. falciparum* liver stages, and warrants further investigation into the control (and timing) of upstream regulators.

Multiple lines of evidence suggest that during malaria, monocytes also have important roles in initiating adaptive responses.^8^, ^44^, ^45^ Previous studies have shown that monocytes increase expression of B‐cell‐activating factor (BAFF) during experimental *P. falciparum* infection with sporozoites,^46^ suggesting monocytes may contribute to B‐cell activation.^47^ In this study, monocyte transcriptional profiling indicated a predominantly MHC class I antigen presentation pathway signature, suggesting monocytes may contribute to the activation of CD8 T cells that have recently been shown to kill *Plasmodium*‐infected reticulocytes^48^ and parasitised erythroblasts in a murine malaria model.^49^ Further, HLA‐DR expression on monocytes during infection was retained, consistent with our previous studies where monocytes retain their ability to upregulate HLA‐DR expression in response to TLR ligands or pRBC during subpatent primary *P. falciparum* infection.^50^ In contrast, during both experimental subpatent infection and clinical malaria, HLA‐DR expression is reduced on classical CD1c^+^ DC,^50^, ^51^ which are typically considered superior antigen‐presenting cells.^52^ Similarly, we have previously reported that clinical malaria drives impaired DC function and increased apoptosis.^51^ Thus, together data support a role of monocytes as important antigen‐presenting cells, in contrast to DCs, for initiation of adaptive T‐ and B‐cell immune responses during malaria.

*Plasmodium falciparum* infection resulted in changes in monocyte composition and phenotype which was sustained for up to 1 month after a first infection. These results suggest that *P. falciparum* may drive ‘innate immune training’,^53^ in which innate immune cells such as monocytes undergo specific changes in their chromatin profiles induced by pathogen stimulation.^53^ Sustained changes seen here include an increased proportion of classical (CD14^+^CD16^−^) monocytes and reduced proportions of non‐classical (CD14^−^CD16^+^) monocytes in the periphery until at least day 45 following infection. In homeostasis, monocytes migrate from the bone marrow and can circulate in the periphery for approximately 1 day.^54^ Subsequently, 90% of monocytes migrate to tissues and become macrophages, while 10% differentiate to intermediate monocytes. The elevated proportion of classical monocytes in the periphery following *P. falciparum* infection may be a consequence of a sustained increase in bone marrow production of monocytes. Consistent with this, we show increased transcription and cell surface expression of CCR2, which mediates monocyte emigration from the bone marrow.^26^ Changes to the composition of monocytes occurred simultaneously with multiple phenotypic changes that were also maintained up to 45 days following infection. These sustained changes are consistent with monocyte training or imprinting.^53^, ^55^ Long‐term effects on monocytes post‐infection have been shown in both Q‐fever^56^ and human *Toxoplasma gondii* infection.^57^ These long‐term effects suggest these infections may influence the transcriptional programme of monocytes and potentially their myeloid progenitors. In support of this notion, heightened monocyte transcriptional responsiveness in Fulani populations infected with *P. falciparum* was suggested to be indicative of genomewide chromatin remodelling.^20^ Future studies investigating parasite‐driven changes to the epigenetic landscape of monocytes following primary infection may be informative.

During clinical malaria, the monocyte transcriptional profile had both similarities (e.g. cell surface signalling and regulation of immune response) and differences (e.g. type I IFN signalling) to that seen during primary subpatent infection. Phagocytosis pathway was one pathway upregulated in both malaria cohorts and is a key function of monocytes in protective immunity.^58^ In primary infection, both transcriptional pathways and cell surface expression of CD64/FcγRI and CD32/FcγRII on monocytes changed significantly and in clinical malaria CD64/FcγRI expression increased. These findings are in contrast with a previous study of Kenyan children with uncomplicated malaria, where monocyte phagocytosis of pRBC was impaired during both symptomatic infection and asymptomatic infection.^16^ Differences between studies may be explained by differences in malaria transmission (perennial in lowland Papua; seasonal in Kenya), age of study participants or technical differences.

In acute clinical malaria, we show children have higher transcriptional activation of monocytes and more DEGs than adults. Despite greater transcriptional activation, the upstream regulators predicted for adults and children were similarly inflammatory, including IFN‐γ in children and IL‐6 and TREM1 (triggering receptor expressed on myeloid cells‐1) in adults. TREM1 is associated with amplifying the inflammatory response in sepsis^59^ and cancer.^60^ Moreover, the overall direction of transcriptional expression remained similar between children and adults, which suggests the magnitude of transcription could be influenced by parasite load, which was higher in children or that adults have an attenuated response as a consequence of prior infection, age and time to presentation, but otherwise similar responses to infection.

In summary, using transcriptional, functional and phenotypic analyses, we reveal monocyte function during primary subpatent infection as dominated by an IFN molecular signature, with increased proportion of activated classical monocytes. Alongside this inflammatory phenotype, monocytes increased IL‐10 production upon re‐exposure to parasite‐infected RBCs, suggesting a role in controlling inflammation upon multiple parasite exposure. Of note, malaria‐naive volunteers maintained phenotypic changes to monocytes up to 1 month post‐infection. Further studies are needed to understand whether these sustained changes are maintained upon repeat infection in previously malaria‐naive volunteers. Differences in transcriptional activation observed between adults and children with clinical malaria warrant further investigation to understand the biological basis driving these differences and the impact on immunity. Our data suggest that monocytes are active and integral immune cells assisting parasite elimination and controlling inflammation in malaria.

## Methods

### Experimental infection using induced blood‐stage malaria (IBSM)

Nineteen volunteers aged 18–31 years (median 23 [IQR 20–26] years, 89% male) consented to participate in a phase Ib clinical trial testing the efficacy of antimalarial drugs. All studies were registered with US NIH ClinicalTrails.gov (ACTRN12613000565741, ACTRN12613001040752, NCT02281344 and NCT03542149). Blood‐stage parasitaemia was initiated by inoculation of 1800 or 2800 parasitised RBCs (pRBC) as previously described.^50^ In brief, healthy malaria‐naive individuals underwent induced blood‐stage malaria inoculation with 1800 or 2800 viable *P. falciparum* 3D7‐pRBCs, and peripheral parasitaemia was measured by qPCR as described previously.^61^ Participants were treated with antimalarial drugs at day 7 or day 8 of infection, when parasitaemia reached median 10, 346 [IQR 4303–19, 681] parasites per mL (Table 1). Blood samples from 19 volunteers (across four independent studies) were collected prior to infection (day 0), at peak infection (day 7/8), post‐infection 15 and 45 days (end of study, EOS) after inoculation (in analyses, these time points are grouped as 0, 7/8, 15 and 45). Blood samples were taken at the same time each day before and during blood‐stage infection. Flow cytometry assays used fresh whole blood and were processed within 2 h of collection (Figure 1a).

### Clinical malaria cohorts

Peripheral blood mononuclear cells (PBMCs) were collected from 15 adults aged 18–38 years (median 30 [IQR 20–38] years; 66% male) and eight children aged 5–13, (median 11 [IQR 8–13] years; 38% male) with acute uncomplicated *P. falciparum* malaria as part of artemisinin combination therapy efficacy studies conducted in southern Papua, Indonesia.^62^ At presentation to the clinic, the median parasitaemia was 2275 [interquartile range IQR = 788–5712] parasites per µL in adults and 7377 [IQR = 2965–13, 644] parasites per µL in children. Gene expression, phenotype and activation of monocytes were assessed in cryopreserved PBMC samples collected prior to commencing treatment, and again 28 days after antimalarial drug treatment and parasite clearance (Figure 1a).

### Ethics approval

IBSM was approved by the Human Research Ethics Committees of QIMR Berghofer Medical Research, NT Department of Health and Menzies School of Health Research. In the clinical malaria cohorts, written informed consent was obtained from participants, with the study approved by the Human Research Ethics Committees of the National Institute of Health Research and Development, Indonesian Ministry of Health (Jakarta, Indonesia), the NT Department of Health and Menzies School of Health Research.

### Monocyte isolation and RNA sequencing

RNA sequencing was performed on paired samples collected prior to and at peak infection from five subjects experimentally infected with *P. falciparum* pRBC and on acute and convalescence samples from adults and children with acute clinical malaria. IBSM and clinical samples were selected based on the availability of PBMCs at paired time points. Isolation and RNA sequencing for IBSM and clinical samples were performed at separate times by different operators. Monocytes were isolated from PBMCs using the Pan Monocyte Isolation Kit (Miltenyi Biotec, Gladbach, Germany). In brief, monocytes were enriched by indirect magnetic labelling for the isolation of untouched monocytes (all subsets), from human PBMCs. Non‐monocytes, such as T cells, NK cells, B cells, dendritic cells and basophils, were indirectly magnetically labelled using a cocktail of biotin‐conjugated antibodies to anti‐biotin microbeads. Conjugated microbeads and untouched monocytes were washed over MAC isolation columns. Purity of isolated monocyte populations was checked using flow cytometry IBSM (baseline; median 90% [IQR = 84–92], peak infection; median 93% [IQR = 88–96]) and clinical cohorts (acute infection 96% [IQR = 87–98], convalescence 94% [IQR = 86–95]). The distribution of subsets in isolated monocytes was comparable to pre‐sorted populations (Supplementary figure 7). Isolated monocytes were resuspended in RNAprotect (Qiagen, Hilden, Germany) and stored immediately at −80 °C. RNA extraction and RNA sequencing of five paired IBSM study participants, eight paired children (clinical malaria) and ten paired adult (clinical malaria) samples were conducted by Macrogen^©^ (Seoul, Korea) using the Illumina TruSeq Stranded mRNA LT Sample Kit and the HiSeq 2500 instrument. Transcriptome data were analysed using a modified version of an existing variant detection pipeline^63^ consisting of software STAR aligner,^64^ samtools,^65^ HTSeq^66^ and DESeq2.^67^ Reads were first aligned to human reference genome GRCh37 using STAR with the gene model set to gencode v19 annotation and quantmode set to TranscriptomeSAM. The alignment files were sorted with samtools and the resultant reads input to HTSeq to generate raw read counts using the union overlap resolution mode. Read counts were input to DESeq2 and a paired analysis performed for all participants with acute infection and baseline or convalescence data.

### Whole‐blood and PBMC monocyte analysis

Monocytes were characterised as lineage (CD3, CD56, CD19)‐negative, HLA‐DR^+^ and by differential expression of CD14 and CD16. In brief, 200 µL of whole blood or 1 million PBMCs were stained with surface antibodies, CD3 (HIT3a, SK7), CD14 (HCD14, M5E2), CD19 (HIB19, SJ25C1), CD56 (HCD56), HLA‐DR (L243), CD11c (B‐Ly6, Bu15), CD16 (3G8), CD86 (2331, IT2.2), CD319 (SLAMF7, 162.1), CD40 (5C3), CD32 (FUN‐2), CD64 (10.1), CCR2 (K036C2), CD206 (15‐2), CXCR4 (12G5) and CD163 (GHI/61), all antibodies were purchased from BD Biosciences (San Jose, CA, USA) or BioLegend (San Diego, CA, USA). For whole blood, RBCs were lysed with FACS lysing solution (BD Biosciences) and resuspended in 2% FCS/PBS to acquire.

### Monocyte stimulation and intracellular cytokine staining (ICS)

Monocyte cytokine production was assessed in 1 mL of fresh whole blood unstimulated or stimulated with TLR agonists; TLR1: Pam3CSK4 100 ng per mL/TLR2: HKLM 10^8^ cells per mL, TLR4: *Escherichia coli* K12 LPS 200 ng mL^−1^ or TLR7: imiquimod 2.5 µg mL^−1^ (Sigma‐Aldrich, St Louis, MO, USA), pRBC or unparasitised RBC (uRBC) prepared as previously described^50^ at 5 × 10^6^/mL. Protein transport inhibitor (Brefeldin A, GolgiPlug, BD Biosciences) was added after 2 h at 37 °C, 5% CO_2_. At 6 h, cells were stained to identify monocytes: lineage [CD3 (HIT3a), CD19 (HIB19), CD56 (HCD56)]^−^ and CD14^+^ (M5E2), and RBCs were lysed with FACS lysing solution (BD Biosciences), washed with 2% FCS/PBS, cells permeabilised with 1× Perm/Wash™ (BD Biosciences) and stained with intracellular anti‐TNF‐α (MAB11), IL‐12/IL‐23p40 (C11.5), IL‐10 (JES3‐9D7) or IgG1 isotype controls (BioLegend). To determine the stimulant‐specific response, spontaneous cytokine production was subtracted from responses to pRBC, uRBC or TLR agonists.

FACS data were acquired using a FACSCanto™ II (BD Biosciences), Gallios™ (Beckman Coulter, Brea, CA, USA) or Fortessa 5 laser (BD Biosciences) and data analysed using Kaluza^®^ 1.3 (Beckman Coulter) or FlowJo version 10.6 (BD Biosciences).

### Statistics

Statistical analyses and graph generation performed using R studio and GraphPad Prism. The Friedman multiple comparisons test was used to compare longitudinal data in the IBSM cohort. For *post hoc* statistics, a pairwise sign test with the Bonferroni correction for multiple comparisons was performed. For clinical cohorts, the Wilcoxon matched‐pairs signed‐rank test was used as paired data. Tests were two‐tailed and considered significant if *P*‐values < 0.05. For pathway enrichment, the online tool STRING version 11^68^ was used to identify GO biological pathways, and for all analysis, an interaction of high confidence was employed (score ≥ 0.7). Ingenuity Pathway Analysis (IPA) (Qiagen, Hilden, Germany) was used for canonical pathway enrichment and predicted upstream regulator analysis using DEGs with a false detection rate (FDR) < 0.05 unless stated otherwise, with no fold‐change cut‐off.

## Conflict of interest

The authors declare no conflict of interest.

## Author contributions

**Jessica R Loughland:** Formal analysis; Investigation; Visualization; Writing‐original draft; Writing‐review & editing. **Tonia Woodberry:** Conceptualization; Funding acquisition; Investigation; Methodology; Supervision; Writing‐review & editing. **Matt Field:** Data curation; Formal analysis; Methodology; Software; Writing‐review & editing. **Dean W Andrew:** Methodology; Writing‐review & editing. **Arya SheelaNair:** Methodology; Writing‐review & editing. **Nicholas L Dooley:** Methodology; Writing‐review & editing. **Kim A Piera:** Methodology; Project administration; Writing‐review & editing. **Fiona H Amante:** Project administration; Writing‐review & editing. **Enny Kenangalem:** Project administration; Supervision; Writing‐review & editing. **Ric N Price:** Funding acquisition; Project administration; Resources; Writing‐review & editing. **Christian R Engwerda:** Funding acquisition; Project administration; Resources; Writing‐review & editing. **Nicholas M Anstey:** Conceptualization; Funding acquisition; Project administration; Resources; Writing‐review & editing. **James S McCarthy:** Funding acquisition; Project administration; Resources; Writing‐review & editing. **Michelle J Boyle:** Funding acquisition; Investigation; Resources; Supervision; Writing‐review & editing. **Gabriela Minigo:** Conceptualization; Investigation; Methodology; Supervision; Writing‐review & editing.

## Supporting information

Supplementary figures 1‐7

Supplementary tables 1‐3
