# Supplementary material for: Transcriptional profiling and immunophenotyping show sustained activation of blood monocytes in subpatent Plasmodium falciparum infection
Source: Clin Transl Immunology. 2020 Jun 18;9(6):e1144. doi: 10.1002/cti2.1144 (PMC7302943; doi:10.1002/cti2.1144)

Transcriptional profiling and immunophenotyping show sustained activation of blood monocytes in subpatent *Plasmodium falciparum* infection.

Jessica R. Loughland[1^], Tonia Woodberry[2¥], Matt Field[3,4], Dean W. Andrew[1], Arya SheelaNair[1], Nicholas L. Dooley[1], Kim A. Piera[2], Fiona H. Amante[2], Enny Kenangalem[5,6], Ric N. Price[2,7,8], Christian R. Engwerda[1], Nicholas M. Anstey[2], James S. McCarthy[1], Michelle J Boyle[1*] and Gabriela Minigo[2,9*]

[1]QIMR Berghofer Medical Research Institute, Brisbane, Australia; [2]Menzies School of Health Research, Darwin, Australia and Charles Darwin University, Darwin, Australia; [3]Australian Institute of Tropical Health and Medicine and Centre for Tropical Bioinformatics and Molecular Biology, James Cook University, Cairns, Australia; [4]John Curtin School of Medical Research, Australian National University, Canberra, Australia; [5]Timika Malaria Research Program, Papuan Health and Community Development Foundation, Timika, Papua, Indonesia; [6]District Health Authority, Timika, Papua, Indonesia; [7]Centre for Tropical Medicine and Global Health, Nuffield Department of Clinical Medicine, University of Oxford, Oxford, UK; [8]Mahidol-Oxford Tropical Medicine Research Unit, Faculty of Tropical Medicine, Mahidol University, Bangkok, Thailand; [9]College of Health and Human Sciences, Charles Darwin University, Darwin, Australia.

¥ Present Address: The University of Newcastle, Australia.
^ Previous Address: Menzies School of Health Research, Darwin, Australia
* These authors contributed equally to this work.

Supplementary Figure 1 Monocyte pathway analysis using STRING after primary *P. falciparum* infection (VIS). Pathway analysis performed using STRING (interactions of high confidence score ≥0.7), GO biological processes ranked by FDR. For analyses DEG input FDR<0.01, n=5 paired samples. FDR= false discovery rate, DEGs= differentially expressed genes.

Supplementary Figure 2 Monocyte TNF and IL-10 production in response to pRBC stimulation at baseline (day 0). The Wilcoxon sign rank test was used to compare paired data, uRBC versus pRBC. Tests were two-tailed and considered significant if *P*-values <0.05. pRBC= parasitized RBC, uRBC= uninfected RBC.

Supplementary Figure 3 Monocyte pathway analysis using STRING in children with acute clinical malaria. (a) Pathway analysis performed using STRING (interactions of high confidence score ≥0.7), GO biological processes ranked by FDR. **(b).** Predicted upstream regulators induced, analysis performed using IPA, red bars indicate activated, blue bars indicate inhibited, Benjamini-Hochberg-adjusted *P*-values, n=8 paired samples. FDR= false discovery rate,

Supplementary Figure 4 Monocyte pathway analysis using STRING in adults with acute clinical malaria. (a) Pathway analysis performed using STRING (interactions of high confidence score ≥0.7), GO biological processes ranked by FDR. **(b)** Predicted upstream regulators induced, analysis performed using IPA, red bars indicate activated, blue bars indicate inhibited, Fishers-exact test used to calculate *P*-values, n=10 paired samples. FDR= false discovery rate.

Supplementary Figure 5. Monocyte phenotype in children and adults with acute clinical malaria. Surface expression of **(a)** CD86 percent gated **(b)** CXCR4 percent gated and **(c)** CD206 (mannose receptor) MFI. Box plots show the 10th-90th percentile, median and interquartile range

for data from all participants. Data points outside of box plots represent patients which were outliers. The Wilcoxon sign rank test was used to compare longitudinal data. Tests were two-tailed and considered significant if *P*-values <0.05. Child cohort (day 0; n=3, day 28 n=3), Adult cohort (day 0; n=9, day 28; n= 11). FSC=forward scatter, SSC=side scatter, MFI= median fluorescence intensity.

Supplementary Figure 6. Representative flow cytometry plots of isolated monocytes. (a) VIS, peak-infection and baseline samples. **(b)** Clinical malaria, acute infection and convalescent samples.

Supplementary Figure 1

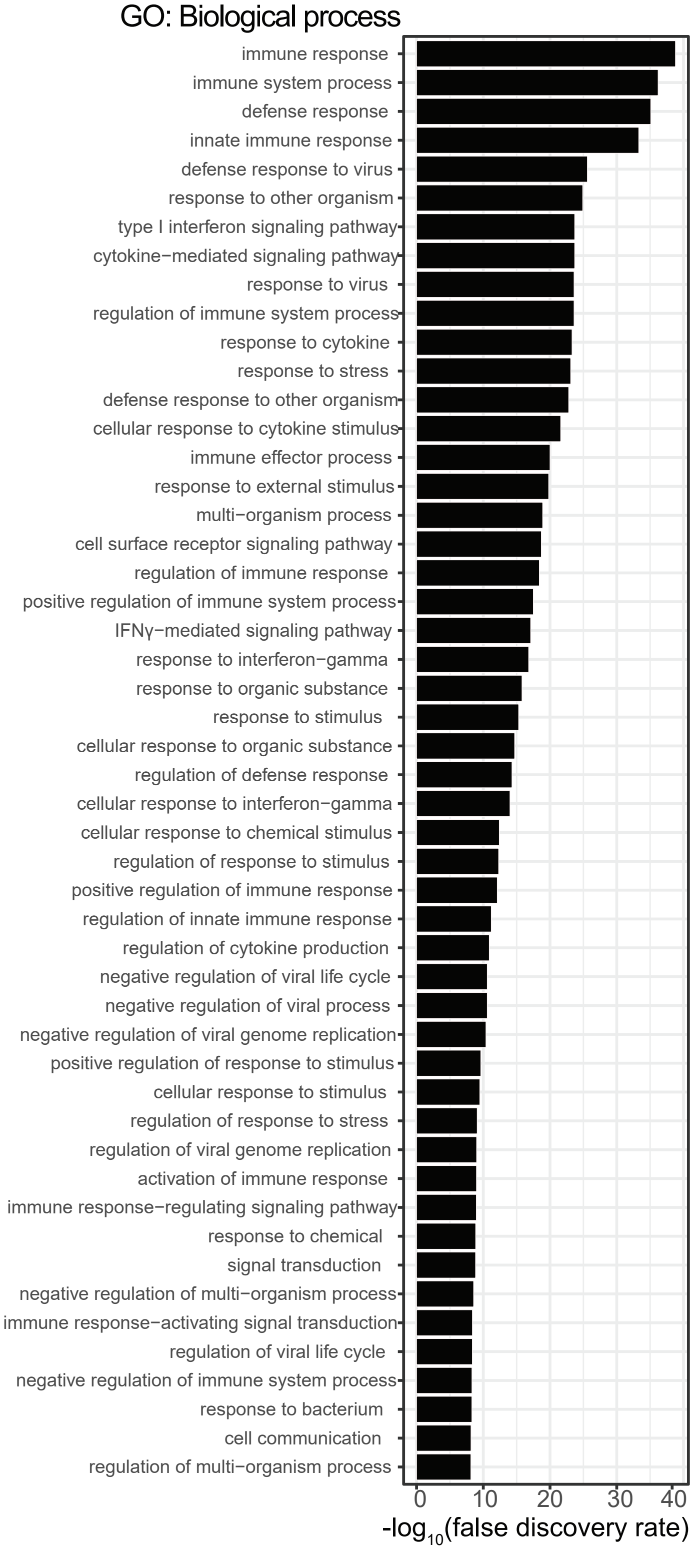

Supplementary Figure 2

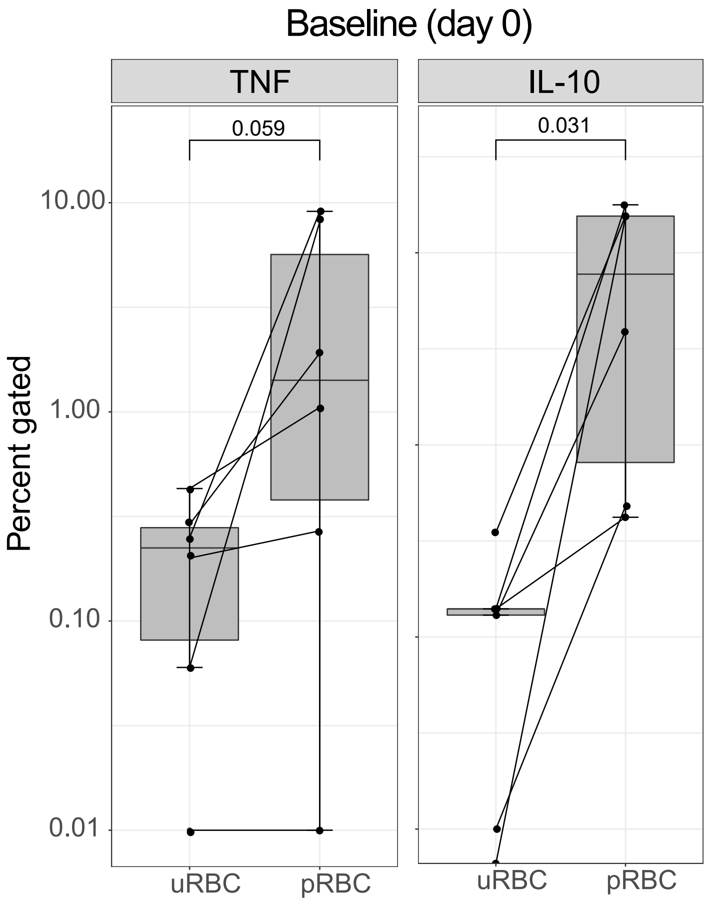

Supplementary Figure 3

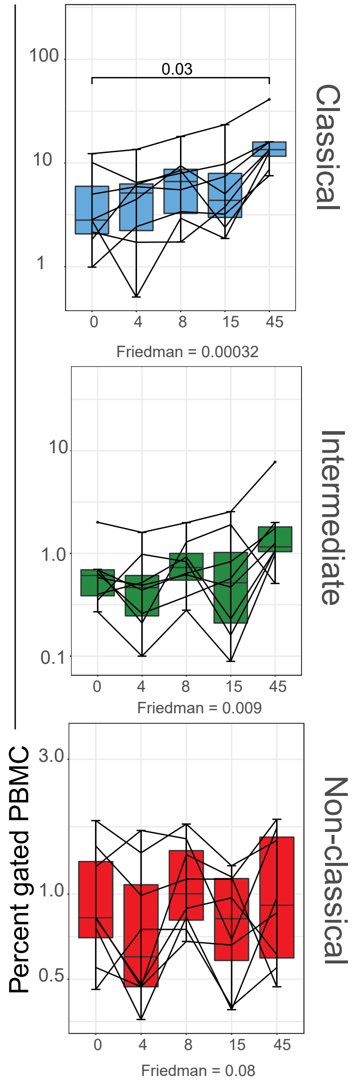

Supplementary Figure 4

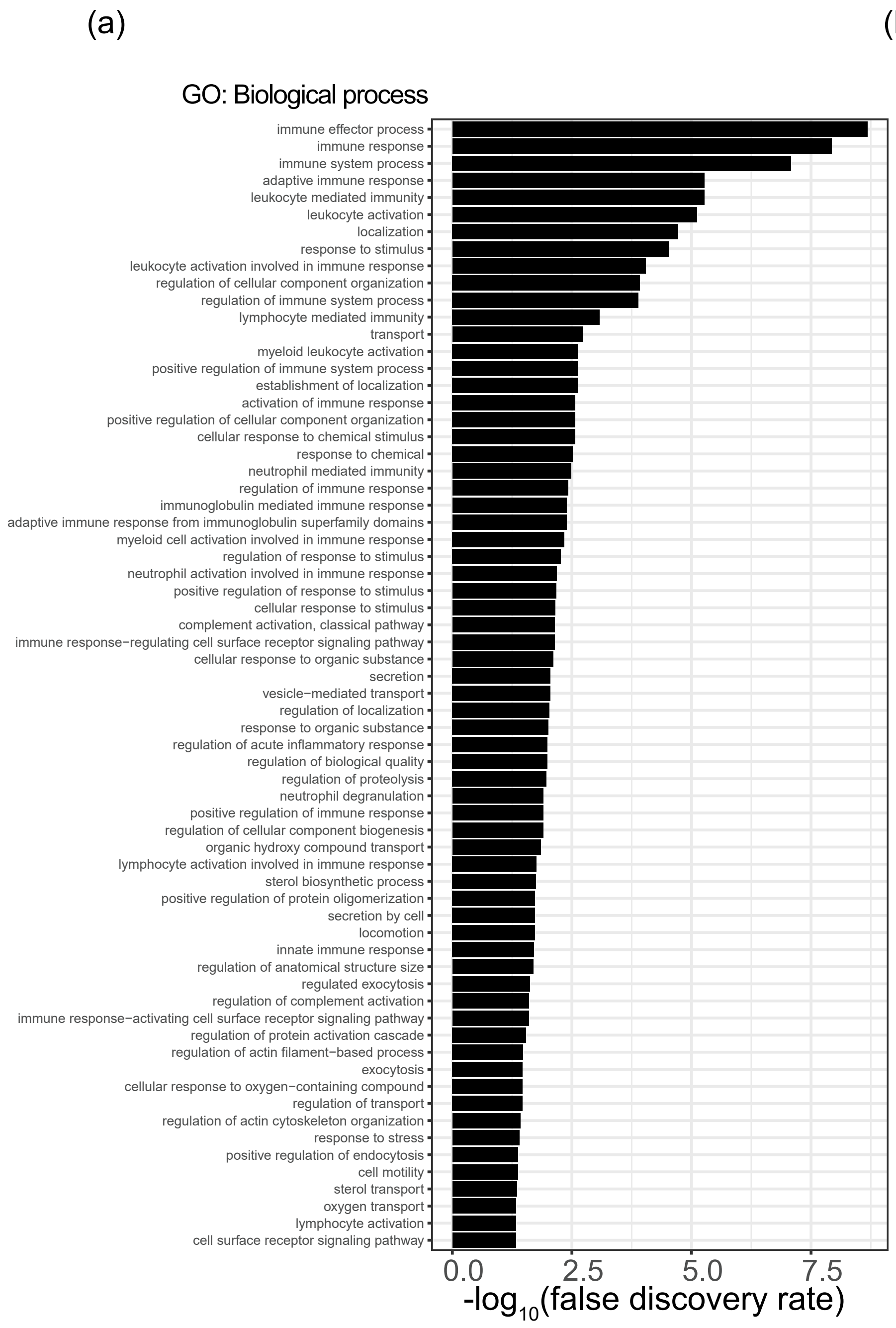

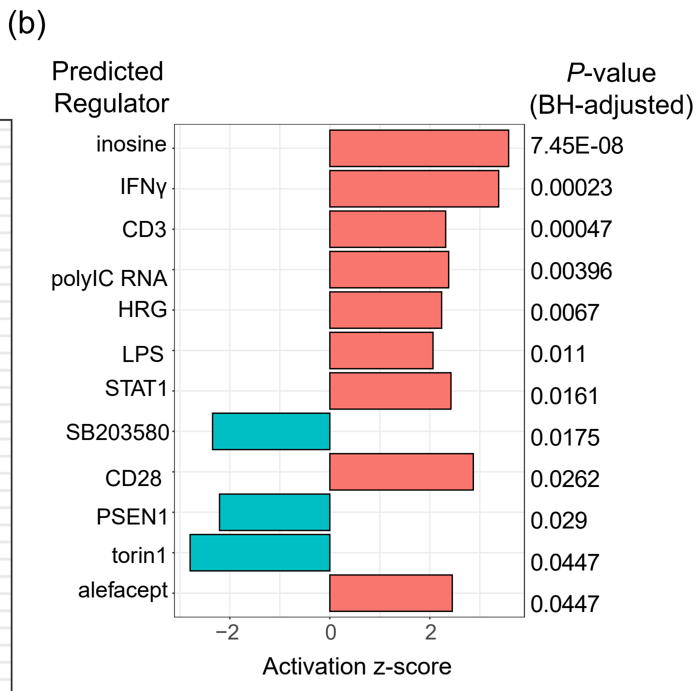

Supplementary Figure 5

(a)

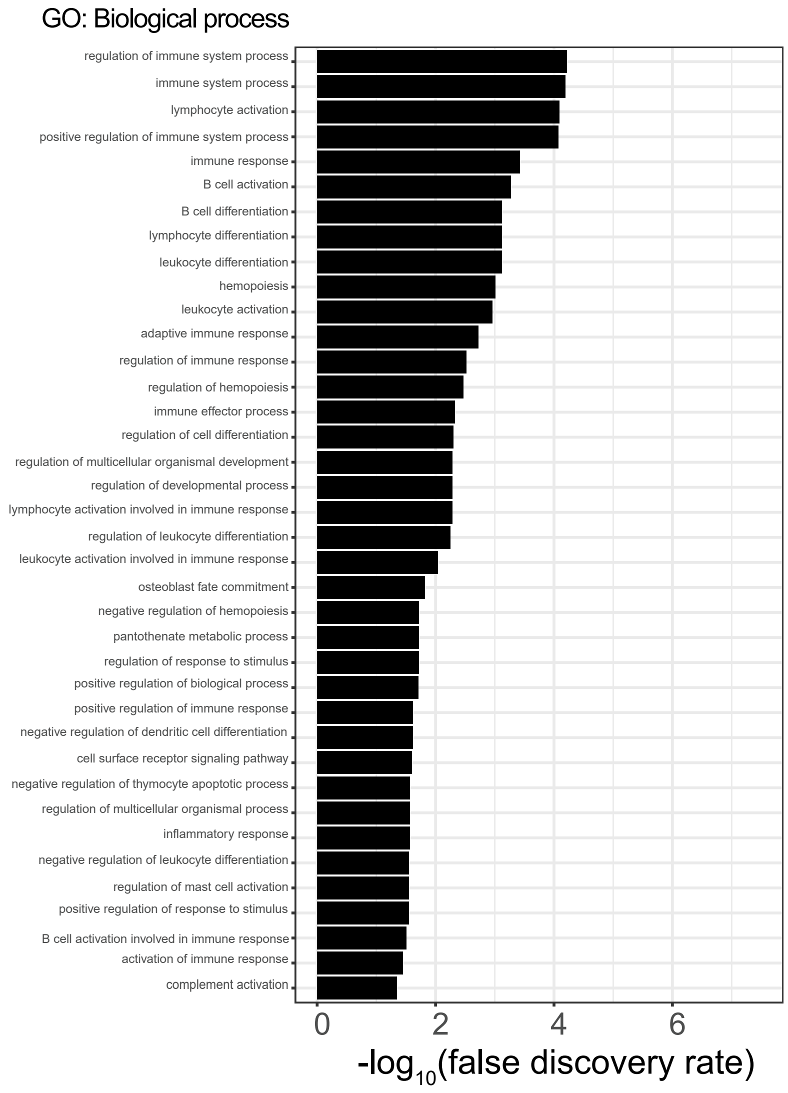

(b)

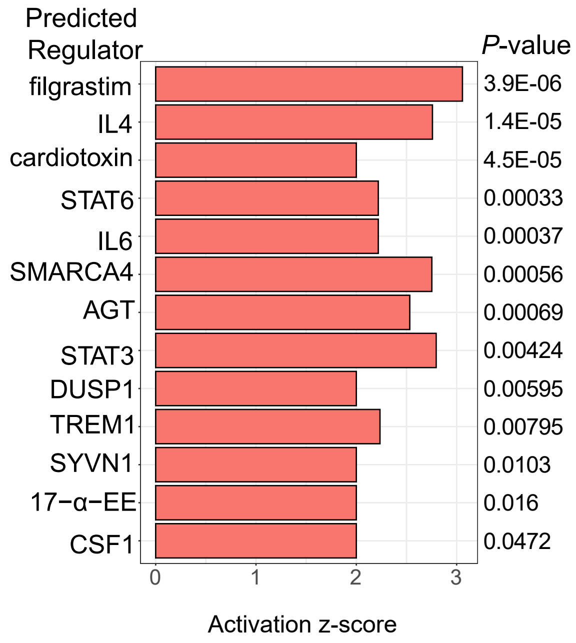

Supplementary Figure 6

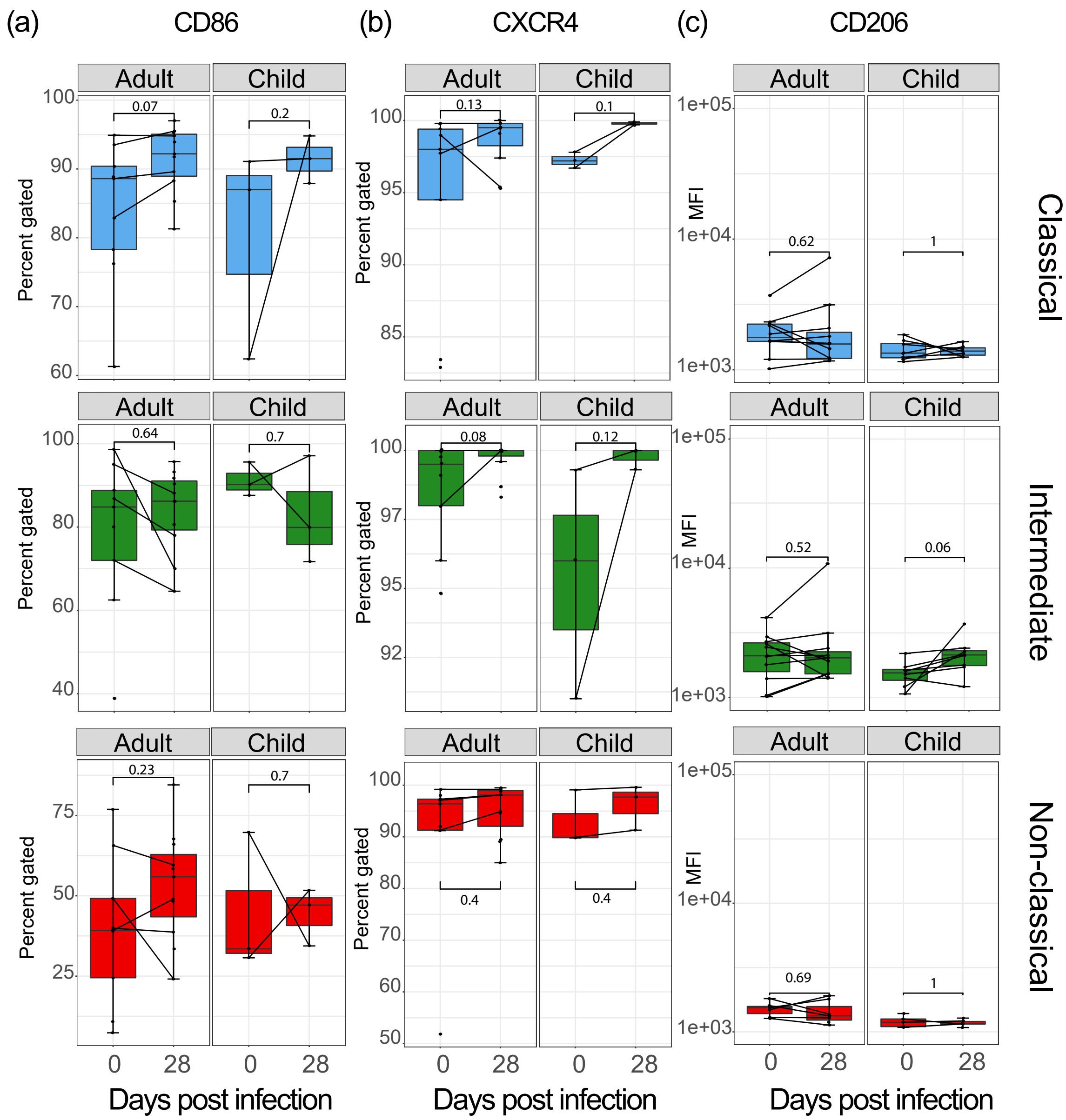

Supplementary Figure 7

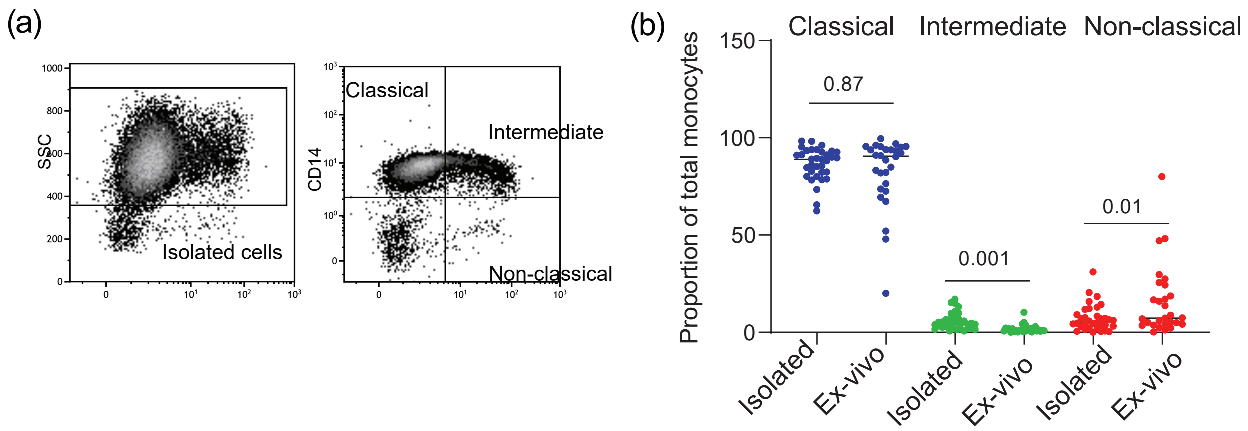

Supplement: Supplementary file 1 — Supplementary figures 1‐7 [file CTI2-9-e1144-s001.pdf]
