# Supplementary material for: Transcriptional profiling and immunophenotyping show sustained activation of blood monocytes in subpatent Plasmodium falciparum infection
Source: Clin Transl Immunology. 2020 Jun 18;9(6):e1144. doi: 10.1002/cti2.1144 (PMC7302943; doi:10.1002/cti2.1144)
Supplement: Supplementary file 2 — Supplementary tables 1‐3 [file CTI2-9-e1144-s002.pdf]

Supplementary Table 1. List of significant DEGs in VIS cohort (FDR<0.05)					
**Ensemble gene ID**	**Gene symbol**	**Ensemble gene ID**	**Gene symbol**	**Ensemble gene ID**	**Gene symbol**
ENSG00000211893	IGHG2	ENSG00000133561	GIMAP6	ENSG00000084733	RAB10
ENSG00000010030	ETV7	ENSG00000149131	SERPING1	ENSG00000172543	CTSW
ENSG00000211659	IGLV3-25	ENSG00000128394	APOBEC3F	ENSG00000163823	CCR1
ENSG00000177409	SAMD9L	ENSG00000161929	SCIMP	ENSG00000156127	BATF
ENSG00000211592	IGKC	ENSG00000132465	JCHAIN	ENSG00000166278	C2
ENSG00000211666	IGLV2-14	ENSG00000168016	TRANK1	ENSG00000197930	ERO1A
ENSG00000163565	IFI16	ENSG00000126351	THRA	ENSG00000182378	PLCXD1
ENSG00000069702	TGFBR3	ENSG00000171219	CDC42BPG	ENSG00000119686	FLVCR2
ENSG00000132109	TRIM21	ENSG00000187164	SHTN1	ENSG00000155506	LARP1
ENSG00000117226	GBP3	ENSG00000165506	DNAAF2	ENSG00000140450	ARRDC4
ENSG00000138755	CXCL9	ENSG00000023171	GRAMD1B	ENSG00000073737	DHRS9
ENSG00000173193	PARP14	ENSG00000185215	TNFAIP2	ENSG00000183458	AC138932.1
ENSG00000113368	LMNB1	ENSG00000124256	ZBP1	ENSG00000256043	CTSO
ENSG00000239855	IGKV1-6	ENSG00000196735	HLA-DQA1	ENSG00000079616	KIF22
ENSG00000111335	OAS2	ENSG00000140749	IGSF6	ENSG00000253882	AC099548.2
ENSG00000163563	MNDA	ENSG00000175550	DRAP1	ENSG00000109063	MYH3
ENSG00000162614	NEXN	ENSG00000163568	AIM2	ENSG00000091106	NLRC4
ENSG00000252010	SCARNA5	ENSG00000108691	CCL2	ENSG00000277443	MARCKS
ENSG00000096968	JAK2	ENSG00000104518	GSDMD	ENSG00000075568	TMEM131
ENSG00000261150	EPPK1	ENSG00000151693	ASAP2	ENSG00000174718	KIAA1551
ENSG00000205413	SAMD9	ENSG00000105967	TFEC	ENSG00000183621	ZNF438
ENSG00000055130	CUL1	ENSG00000283537	AC073264.3	ENSG00000074181	NOTCH3
ENSG00000025708	TYMP	ENSG00000153283	CD96	ENSG00000085978	ATG16L1
ENSG00000125347	IRF1	ENSG00000132963	POMP	ENSG00000058866	DGKG
ENSG00000198604	BAZ1A	ENSG00000122877	EGR2	ENSG00000085265	FCN1
ENSG00000158517	NCF1	ENSG00000152778	IFIT5	ENSG00000211689	TRGC1
ENSG00000185745	IFIT1	ENSG00000169136	ATF5	ENSG00000165355	FBXO33
ENSG00000128284	APOL3	ENSG00000166750	SLFN5	ENSG00000015285	WAS
ENSG00000239951	IGKV3-20	ENSG00000126838	PZP	ENSG00000250138	AC139495.3
ENSG00000211943	IGHV3-15	ENSG00000111181	SLC6A12	ENSG00000214872	SMTNL1
ENSG00000212232	SNORD17	ENSG00000139192	TAPBPL	ENSG00000198938	MT-CO3
ENSG00000211899	IGHM	ENSG00000125826	RBCK1	ENSG00000137752	CASP1
ENSG00000196664	TLR7	ENSG00000008710	PKD1	ENSG00000160888	IER2
ENSG00000115523	GNLY	ENSG00000151948	GLT1D1	ENSG00000112110	MRPL18
ENSG00000177989	ODF3B	ENSG00000164442	CITED2	ENSG00000167202	TBC1D2B
ENSG00000198959	TGM2	ENSG00000137959	IFI44L	ENSG00000179899	PHC1P1
ENSG00000182487	NCF1B	ENSG00000106565	TMEM176B	ENSG00000172086	KRCC1
ENSG00000171115	GIMAP8	ENSG00000277072	STAG3L2	ENSG00000197272	IL27
ENSG00000123609	NMI	ENSG00000073331	ALPK1	ENSG00000185130	HIST1H2BL

ENSG00000165178	NCF1C	ENSG00000154451	GBP5	ENSG00000111269	CREBL2
ENSG00000146072	TNFRSF21	ENSG00000130787	HIP1R	ENSG00000204516	MICB
ENSG00000115415	STAT1	ENSG00000106605	BLVRA	ENSG00000249855	EEF1A1P19
ENSG00000135899	SP110	ENSG00000138660	AP1AR	ENSG00000137393	RNF144B
ENSG00000108387	SEPTIN4	ENSG00000002549	LAP3	ENSG00000248477	AC139495.1
ENSG00000170581	STAT2	ENSG00000115091	ACTR3	ENSG00000185507	IRF7
ENSG00000184730	APOBR	ENSG00000119922	IFIT2	ENSG00000153563	CD8A
ENSG00000135636	DYSF	ENSG00000221963	APOL6	ENSG00000171503	ETFDH
ENSG00000183091	NEB	ENSG00000013364	MVP	ENSG00000198252	STYX
ENSG00000137801	THBS1	ENSG00000278196	IGLV2-8	ENSG00000107651	SEC23IP
ENSG00000133106	EPSTI1	ENSG00000198682	PAPSS2	ENSG00000162894	FCMR
ENSG00000133313	CNDP2	ENSG00000169245	CXCL10	ENSG00000067369	TP53BP1
ENSG00000243466	IGKV1-5	ENSG00000115392	FANCL	ENSG00000250687	AC146944.2
ENSG00000174125	TLR1	ENSG00000118689	FOXO3	ENSG00000151490	PTPRO
ENSG00000132530	XAF1	ENSG00000166532	RIMKLB	ENSG00000100100	PIK3IP1
ENSG00000101017	CD40	ENSG00000265531	FCGR1CP	ENSG00000070190	DAPP1
ENSG00000253755	IGHGP	ENSG00000198087	CD2AP	ENSG00000157693	TMEM268
ENSG00000188282	RUFY4	ENSG00000284669	AC092053.3	ENSG00000135842	FAM129A
ENSG00000179094	PER1	ENSG00000026103	FAS	ENSG00000137312	FLOT1
ENSG00000272821	U62317.2	ENSG00000111331	OAS3	ENSG00000106780	MEGF9
ENSG00000179144	GIMAP7	ENSG00000138385	SSB	ENSG00000121671	CRY2
ENSG00000068079	IFI35	ENSG00000059378	PARP12	ENSG00000130348	QRSL1
ENSG00000135148	TRAFD1	ENSG00000187608	ISG15	ENSG00000107566	ERLIN1
ENSG00000116663	FBXO6	ENSG00000279602	AC109326.1	ENSG00000171051	FPR1
ENSG00000150907	FOXO1	ENSG00000112367	FIG4	ENSG00000123700	KCNJ2
ENSG00000178537	SLC25A20	ENSG00000165949	IFI27	ENSG00000158874	APOA2
ENSG00000198814	GK	ENSG00000185339	TCN2	ENSG00000170542	SERPINB9
ENSG00000145287	PLAC8	ENSG00000027075	PRKCH	ENSG00000156587	UBE2L6
ENSG00000031081	ARHGAP31	ENSG00000093072	ADA2	ENSG00000273897	AC211476.3
ENSG00000133574	GIMAP4	ENSG00000158714	SLAMF8	ENSG00000112096	SOD2
ENSG00000134755	DSC2	ENSG00000085449	WDFY1	ENSG00000108055	SMC3
ENSG00000028116	VRK2	ENSG00000205583	STAG3L1	ENSG00000092531	SNAP23
ENSG00000198821	CD247	ENSG00000185950	IRS2	ENSG00000163220	S100A9
ENSG00000132274	TRIM22	ENSG00000121966	CXCR4	ENSG00000171316	CHD7
ENSG00000054654	SYNE2	ENSG00000211685	IGLC7	ENSG00000173369	C1QB
ENSG00000041357	PSMA4	ENSG00000172183	ISG20	ENSG00000030582	GRN
ENSG00000109906	ZBTB16	ENSG00000127954	STEAP4	ENSG00000163739	CXCL1
ENSG00000020577	SAMD4A	ENSG00000196954	CASP4	ENSG00000157557	ETS2
ENSG00000185499	MUC1	ENSG00000065911	MTHFD2	ENSG00000103657	HERC1
ENSG00000157514	TSC22D3	ENSG00000107554	DNMBP	ENSG00000159228	CBR1
ENSG00000140464	PML	ENSG00000131979	GCH1	ENSG00000239521	CASTOR3

ENSG00000082074	FYB1	ENSG00000117054	ACADM	ENSG00000110721	CHKA
ENSG00000114127	XRN1	ENSG00000185404	SP140L	ENSG00000168685	IL7R
ENSG00000013374	NUB1	ENSG00000137965	IFI44	ENSG00000120217	CD274
ENSG00000145349	CAMK2D	ENSG00000204397	CARD16	ENSG00000112419	PHACTR2
ENSG00000163840	DTX3L	ENSG00000150337	FCGR1A	ENSG00000151725	CENPU
ENSG00000211966	IGHV5-51	ENSG00000111452	ADGRD1	ENSG00000258581	AL157871.3
ENSG00000200087	SNORA73B	ENSG00000239713	APOBEC3G	ENSG00000162654	GBP4
ENSG00000152766	ANKRD22	ENSG00000138246	DNAJC13	ENSG00000171204	TMEM126B
ENSG00000121807	CCR2	ENSG00000173110	HSPA6	ENSG00000104805	NUCB1
ENSG00000060491	OGFR	ENSG00000130303	BST2	ENSG00000047634	SCML1
ENSG00000135218	CD36	ENSG00000129515	SNX6	ENSG00000115594	IL1R1
ENSG00000160932	LY6E	ENSG00000227827	AC138969.1	ENSG00000068120	COASY
ENSG00000100342	APOL1	ENSG00000205746	AC126755.1	ENSG00000064607	SUGP2
ENSG00000019169	MARCO	ENSG00000182511	FES	ENSG00000163644	PPM1K
ENSG00000108679	LGALS3BP	ENSG00000157601	MX1	ENSG00000130813	C19orf66
ENSG00000188404	SELL	ENSG00000134574	DDB2	ENSG00000136874	STX17
ENSG00000089127	OAS1	ENSG00000139725	RHOF	ENSG00000198771	RCSD1
ENSG00000076555	ACACB	ENSG00000249437	NAIP	ENSG00000198886	MT-ND4
ENSG00000229859	PGA3	ENSG00000122694	GLIPR2	ENSG00000088992	TESC
ENSG00000263069	AC124319.2	ENSG00000146070	PLA2G7	ENSG00000155097	ATP6V1C1
ENSG00000101916	TLR8	ENSG00000229183	PGA4	ENSG00000197405	C5AR1
ENSG00000115590	IL1R2	ENSG00000179933	C14orf119	ENSG00000188559	RALGAPA2
ENSG00000204267	TAP2	ENSG00000206503	HLA-A	ENSG00000225684	FAM225B
ENSG00000148429	USP6NL	ENSG00000175899	A2M	ENSG00000213512	GBP7
ENSG00000121858	TNFSF10	ENSG00000085840	ORC1	ENSG00000109046	WSB1
ENSG00000145113	MUC4	ENSG00000137767	SQOR	ENSG00000204525	HLA-C
ENSG00000092010	PSME1	ENSG00000026950	BTN3A1	ENSG00000137193	PIM1
ENSG00000155307	SAMSN1	ENSG00000139687	RB1	ENSG00000231528	FAM225A
ENSG00000242371	IGKV1-39	ENSG00000178146	AL672207.1	ENSG00000196743	GM2A
ENSG00000140853	NLRC5	ENSG00000122035	RASL11A	ENSG00000142089	IFITM3
ENSG00000225492	GBP1P1	ENSG00000140750	ARHGAP17	ENSG00000100883	SRP54
ENSG00000151014	NOCT	ENSG00000113448	PDE4D	ENSG00000136156	ITM2B
ENSG00000267519	AC020916.1	ENSG00000081041	CXCL2	ENSG00000213928	IRF9
ENSG00000234518	PTGES3P1	ENSG00000211898	IGHD	ENSG00000115904	SOS1
ENSG00000134326	CMPK2	ENSG00000116337	AMPD2	ENSG00000236567	TCF3P1
ENSG00000211648	IGLV1-47	ENSG00000211677	IGLC2	ENSG00000185187	SIGIRR
ENSG00000107201	DDX58	ENSG00000123908	AGO2	ENSG00000130724	CHMP2A
ENSG00000225131	PSME2P2	ENSG00000111859	NEDD9	ENSG00000136048	DRAM1
ENSG00000138496	PARP9	ENSG00000178685	PARP10	ENSG00000165312	OTUD1
ENSG00000240445	FOXO3B	ENSG00000165806	CASP7	ENSG00000115232	ITGA4
ENSG00000151012	SLC7A11	ENSG00000101347	SAMHD1	ENSG00000166033	HTRA1

ENSG00000166801	FAM111A	ENSG00000204592	HLA-E	ENSG00000205336	ADGRG1
ENSG00000086065	CHMP5	ENSG00000164715	LMTK2	ENSG00000143554	SLC27A3
ENSG00000023902	PLEKHO1	ENSG00000254681	PKD1P5	ENSG00000096996	IL12RB1
ENSG00000100220	RTCB	ENSG00000111752	PHC1	ENSG00000143226	FCGR2A
ENSG00000168310	IRF2	ENSG00000185482	STAC3	ENSG00000164855	TMEM184A
ENSG00000251546	IGKV1D-39	ENSG00000181381	DDX60L	ENSG00000211640	IGLV6-57
ENSG00000175866	BAIAP2	ENSG00000182923	CEP63	ENSG00000264229	RNU4ATAC
ENSG00000171049	FPR2	ENSG00000137364	TPMT	ENSG00000105939	ZC3HAV1
ENSG00000167851	CD300A	ENSG00000179978	AC140134.1	ENSG00000196873	CBWD3
ENSG00000165168	CYBB	ENSG00000100911	PSME2	ENSG00000143376	SNX27
ENSG00000154479	CCDC173	ENSG00000174353	STAG3L3	ENSG00000258521	AL157871.2
ENSG00000204642	HLA-F	ENSG00000211897	IGHG3	ENSG00000142599	RERE
ENSG00000182541	LIMK2	ENSG00000243156	MICAL3	ENSG00000204577	LILRB3
ENSG00000152229	PSTPIP2	ENSG00000168394	TAP1	ENSG00000240065	PSMB9
ENSG00000211653	IGLV1-40	ENSG00000132122	SPATA6	ENSG00000166710	B2M
ENSG00000155657	TTN	ENSG00000103642	LACTB	ENSG00000212195	RF00012
ENSG00000115267	IFIH1	ENSG00000102081	FMR1	ENSG00000131196	NFATC1
ENSG00000111801	BTN3A3	ENSG00000070961	ATP2B1	ENSG00000168209	DDIT4
ENSG00000204264	PSMB8	ENSG00000108424	KPNB1	ENSG00000244257	PKD1P1
ENSG00000119917	IFIT3	ENSG00000256713	PGA5	ENSG00000115525	ST3GAL5
ENSG00000211655	IGLV1-36	ENSG00000162645	GBP2	ENSG00000198237	AC131392.1
ENSG00000141232	TOB1	ENSG00000134954	ETS1	ENSG00000166527	CLEC4D
ENSG00000144848	ATG3	ENSG00000003400	CASP10	ENSG00000138646	HERC5
ENSG00000267293	AC012569.1	ENSG00000166889	PATL1	ENSG00000166068	SPRED1
ENSG00000172785	CBWD1	ENSG00000186470	BTN3A2	ENSG00000173221	GLRX
ENSG00000117228	GBP1	ENSG00000172936	MYD88	ENSG00000134001	EIF2S1
ENSG00000198851	CD3E	ENSG00000179388	EGR3	ENSG00000135828	RNASEL
ENSG00000180644	PRF1	ENSG00000198019	FCGR1B	ENSG00000185697	MYBL1
ENSG00000130589	HELZ2	ENSG00000100918	REC8	ENSG00000134061	CD180
ENSG00000105402	NAPA	ENSG00000123200	ZC3H13	ENSG00000110852	CLEC2B
ENSG00000136630	HLX	ENSG00000146433	TMEM181	ENSG00000224041	IGKV3D-15
ENSG00000188906	LRRK2	ENSG00000136682	CBWD2	ENSG00000169994	MYO7B
ENSG00000120738	EGR1	ENSG00000034053	APBA2	ENSG00000092964	DPYSL2
ENSG00000160710	ADAR	ENSG00000064932	SBNO2	ENSG00000184432	COPB2
ENSG00000102524	TNFSF13B	ENSG00000104325	DECR1	ENSG00000049759	NEDD4L
ENSG00000185477	GPRIN3	ENSG00000108771	DHX58	ENSG00000211625	IGKV3D-20
ENSG00000113369	ARRDC3	ENSG00000153029	MR1	ENSG00000129450	SIGLEC9
ENSG00000179583	CIITA	ENSG00000238741	SCARNA7	ENSG00000105875	WDR91
ENSG00000004468	CD38	ENSG00000124181	PLCG1	ENSG00000132694	ARHGEF11
ENSG00000086300	SNX10	ENSG00000146535	GNA12	ENSG00000198712	MT-CO2
ENSG00000079263	SP140	ENSG00000155363	MOV10	ENSG00000205220	PSMB10

ENSG00000170873	MTSS1	ENSG00000184979	USP18	ENSG00000232882	PHKA1P1
ENSG00000204261	PSMB8-AS1	ENSG00000125363	AMELX	ENSG00000270379	HEATR9
ENSG00000197536	C5orf56	ENSG00000186818	LILRB4	ENSG00000134470	IL15RA
ENSG00000107742	SPOCK2	ENSG00000251634	AC145138.1	ENSG00000162437	RAVER2
ENSG00000112079	STK38	ENSG00000229023	AC067945.1	ENSG00000108819	PPP1R9B
ENSG00000124357	NAGK	ENSG00000174749	FAM241A		
ENSG00000165029	ABCA1	ENSG00000152223	EPG5		
ENSG00000130340	SNX9	ENSG00000150540	HNMT		
ENSG00000121210	TMEM131L	ENSG00000022556	NLRP2		

**Supplementary Table 2. List of significant DEGs in the Child acute malaria cohort (FDR<0.05)**					
**Ensemble gene ID**	**Gene Symbol**	**Ensemble gene ID**	**Gene Symbol**	**Ensemble gene ID**	**Gene Symbol**
ENSG00000138119	MYOF	ENSG00000097033	SH3GLB1	ENSG00000103569	AQP9
ENSG00000135424	ITGA7	ENSG00000168329	CX3CR1	ENSG00000162433	AK4
ENSG00000169994	MYO7B	ENSG00000167601	AXL	ENSG00000180644	PRF1
ENSG00000275395	FCGBP	ENSG00000166578	IQCD	ENSG00000137500	CCDC90B
ENSG00000122591	FAM126A	ENSG00000104381	GDAP1	ENSG00000136754	ABI1
ENSG00000197530	MIB2	ENSG00000133789	SWAP70	ENSG00000197540	GZMM
ENSG00000197993	KEL	ENSG00000244734	HBB	ENSG00000113263	ITK
ENSG00000125144	MT1G	ENSG00000088387	DOCK9	ENSG00000280138	AC027290.2
ENSG00000087237	CETP	ENSG00000260231	KDM7A-DT	ENSG00000205018	AC092384.1
ENSG00000120885	CLU	ENSG00000142173	COL6A2	ENSG00000224712	NPIPA3
ENSG00000211696	TRGV8	ENSG00000212195	RF00012	ENSG00000078589	P2RY10
ENSG00000100427	MLC1	ENSG00000135677	GNS	ENSG00000280407	AC132872.4
ENSG00000198719	DLL1	ENSG00000260691	ANKRD20A1	ENSG00000185499	MUC1
ENSG00000137767	SQOR	ENSG00000134480	CCNH	ENSG00000013364	MVP
ENSG00000101695	RNF125	ENSG00000129255	MPDU1	ENSG00000267749	AC092068.3
ENSG00000134575	ACP2	ENSG00000196839	ADA	ENSG00000203804	ADAMTSL4-AS1
ENSG00000183914	DNAH2	ENSG00000126226	PCID2	ENSG00000174125	TLR1
ENSG00000006025	OSBPL7	ENSG00000166278	C2	ENSG00000277610	RNVU1-4
ENSG00000169413	RNASE6	ENSG00000159640	ACE	ENSG00000135473	PAN2
ENSG00000101412	E2F1	ENSG00000129484	PARP2	ENSG00000157168	NRG1
ENSG00000169715	MT1E	ENSG00000162804	SNED1	ENSG00000160856	FCRL3
ENSG00000166428	PLD4	ENSG00000160285	LSS	ENSG00000092841	MYL6
ENSG00000135749	PCNX2	ENSG00000088179	PTPN4	ENSG00000124733	MEA1
ENSG00000105220	GPI	ENSG00000086288	NME8	ENSG00000196092	PAX5
ENSG00000166446	CDYL2	ENSG00000117308	GALE	ENSG00000113108	APBB3
ENSG00000143603	KCNN3	ENSG00000273338	AC103591.3	ENSG00000184983	NDUFA6
ENSG00000088280	ASAP3	ENSG00000136826	KLF4	ENSG00000169504	CLIC4
ENSG00000026751	SLAMF7	ENSG00000148154	UGCG	ENSG00000206530	CFAP44

ENSG00000173372	C1QA	ENSG00000204136	GGTA1P	ENSG00000170074	FAM153A
ENSG00000158481	CD1C	ENSG00000186815	TPCN1	ENSG00000204406	MBD5
ENSG00000007968	E2F2	ENSG00000175048	ZDHHC14	ENSG00000159128	IFNGR2
ENSG00000160307	S100B	ENSG00000119541	VPS4B	ENSG00000106789	CORO2A
ENSG00000079482	OPHN1	ENSG00000167613	LAIR1	ENSG00000087589	CASS4
ENSG00000267519	AC020916.1	ENSG00000081923	ATP8B1	ENSG00000133316	WDR74
ENSG00000125746	EML2	ENSG00000128923	MINDY2	ENSG00000103769	RAB11A
ENSG00000146386	ABRACL	ENSG00000205809	KLRC2	ENSG00000011201	ANOS1
ENSG00000213934	HBG1	ENSG00000163735	CXCL5	ENSG00000185897	FFAR3
ENSG00000164733	CTSB	ENSG00000105185	PDCD5	ENSG00000157593	SLC35B2
ENSG00000110958	PTGES3	ENSG00000167106	FAM102A	ENSG00000101189	MRGBP
ENSG00000109062	SLC9A3R1	ENSG00000108405	P2RX1	ENSG00000080031	PTPRH
ENSG00000050393	MCUR1	ENSG00000179542	SLITRK4	ENSG00000138613	APH1B
ENSG00000149177	PTPRJ	ENSG00000109684	CLNK	ENSG00000151208	DLG5
ENSG00000137193	PIM1	ENSG00000174099	MSRB3	ENSG00000106733	NMRK1
ENSG00000152766	ANKRD22	ENSG00000135404	CD63	ENSG00000099985	OSM
ENSG00000158869	FCER1G	ENSG00000274210	RF00003	ENSG00000137185	ZSCAN9
ENSG00000275538	RNVU1-19	ENSG00000086666	ZFAND6	ENSG00000140398	NEIL1
ENSG00000170439	METTL7B	ENSG00000134545	KLRC1	ENSG00000176261	ZBTB8OS
ENSG00000230989	HSBP1	ENSG00000001167	NFYA	ENSG00000260229	PPIAP51
ENSG00000196776	CD47	ENSG00000163520	FBLN2	ENSG00000128159	TUBGCP6
ENSG00000143653	SCCPDH	ENSG00000158186	MRAS	ENSG00000127152	BCL11B
ENSG00000130600	H19	ENSG00000100307	CBX7	ENSG00000204815	TTC25
ENSG00000175455	CCDC14	ENSG00000155366	RHOC	ENSG00000196796	NPIPB10P
ENSG00000234369	TATDN1P1	ENSG00000090447	TFAP4	ENSG00000185163	DDX51
ENSG00000182162	P2RY8	ENSG00000117450	PRDX1	ENSG00000005249	PRKAR2B
ENSG00000101856	PGRMC1	ENSG00000131174	COX7B	ENSG00000227191	TRGC2
ENSG00000162591	MEGF6	ENSG00000105509	HAS1	ENSG00000284669	AC092053.3
ENSG00000173369	C1QB	ENSG00000151136	BTBD11	ENSG00000117425	PTCH2
ENSG00000073605	GSDMB	ENSG00000167553	TUBA1C	ENSG00000136732	GYPC
ENSG00000030066	NUP160	ENSG00000163568	AIM2	ENSG00000223901	AP001469.1
ENSG00000150687	PRSS23	ENSG00000103064	SLC7A6	ENSG00000125257	ABCC4
ENSG00000075624	ACTB	ENSG00000136167	LCP1	ENSG00000177098	SCN4B
ENSG00000151623	NR3C2	ENSG00000111229	ARPC3	ENSG00000156886	ITGAD
ENSG00000168026	TTC21A	ENSG00000140563	MCTP2	ENSG00000186174	BCL9L
ENSG00000265531	FCGR1CP	ENSG00000157045	NTAN1	ENSG00000180611	MB21D2
ENSG00000034053	APBA2	ENSG00000158488	CD1E	ENSG00000139537	CCDC65
ENSG00000109065	NAT9	ENSG00000147133	TAF1	ENSG00000167600	CYP2S1
ENSG00000138600	SPPL2A	ENSG00000123416	TUBA1B	ENSG00000112290	WASF1
ENSG00000113070	HBEGF	ENSG00000104177	MYEF2	ENSG00000141698	NT5C3B
ENSG00000104814	MAP4K1	ENSG00000142512	SIGLEC10	ENSG00000003056	M6PR

ENSG00000158470	B4GALT5	ENSG00000147400	CETN2	ENSG00000171502	COL24A1
ENSG00000147459	DOCK5	ENSG00000272419	LINC01145	ENSG00000198892	SHISA4
ENSG00000164292	RHOBTB3	ENSG00000144218	AFF3	ENSG00000196154	S100A4
ENSG00000075142	SRI	ENSG00000155657	TTN	ENSG00000240563	L1TD1
ENSG00000138821	SLC39A8	ENSG00000136153	LMO7	ENSG00000183148	ANKRD20A2
ENSG00000116962	NID1	ENSG00000091592	NLRP1	ENSG00000112695	COX7A2
ENSG00000114209	PDCD10	ENSG00000214544	GTF2IRD2P1	ENSG00000196169	KIF19
ENSG00000123240	OPTN	ENSG00000168246	UBTD2	ENSG00000283674	AC068587.4
ENSG00000214967	NPIPA7	ENSG00000127526	SLC35E1	ENSG00000176658	MYO1D
ENSG00000159189	C1QC	ENSG00000133687	TMTC1	ENSG00000188735	TMEM120B
ENSG00000274102	OR4M2	ENSG00000162512	SDC3	ENSG00000188599	NPIPP1
ENSG00000109066	TMEM104	ENSG00000027075	PRKCH	ENSG00000188886	ASTL
ENSG00000165914	TTC7B	ENSG00000139970	RTN1	ENSG00000188185	LINC00265
ENSG00000163687	DNASE1L3	ENSG00000113552	GNPDA1	ENSG00000120137	PANK3
ENSG00000168899	VAMP5	ENSG00000123609	NMI	ENSG00000164919	COX6C
ENSG00000185697	MYBL1	ENSG00000189043	NDUFA4	ENSG00000137714	FDX1
ENSG00000070814	TCOF1	ENSG00000223705	NSUN5P1	ENSG00000102316	MAGED2
ENSG00000177556	ATOX1	ENSG00000165685	TMEM52B	ENSG00000219928	AL161787.1
ENSG00000198019	FCGR1B	ENSG00000135596	MICAL1	ENSG00000025434	NR1H3
ENSG00000198156	NPIPB6	ENSG00000168702	LRP1B	ENSG00000180720	CHRM4
ENSG00000150337	FCGR1A	ENSG00000168763	CNNM3	ENSG00000064651	SLC12A2
ENSG00000161509	GRIN2C	ENSG00000134061	CD180	ENSG00000225492	GBP1P1
ENSG00000143633	C1orf131	ENSG00000184613	NELL2	ENSG00000155849	ELMO1
ENSG00000077549	CAPZB	ENSG00000276216	AC245014.3	ENSG00000244509	APOBEC3C
ENSG00000116133	DHCR24	ENSG00000196562	SULF2	ENSG00000145730	PAM
ENSG00000122565	CBX3	ENSG00000105639	JAK3	ENSG00000164741	DLC1
ENSG00000167552	TUBA1A	ENSG00000137726	FXYD6	ENSG00000264229	RNU4ATAC
ENSG00000183762	KREMEN1	ENSG00000165795	NDRG2	ENSG00000172594	SMPDL3A
ENSG00000160213	CSTB	ENSG00000196230	TUBB	ENSG00000029153	ARNTL2
ENSG00000108950	FAM20A	ENSG00000139718	SETD1B	ENSG00000206828	RF00003
ENSG00000007402	CACNA2D2	ENSG00000157445	CACNA2D3	ENSG00000197785	ATAD3A
ENSG00000167280	ENGASE	ENSG00000048028	USP28	ENSG00000197860	SGTB
ENSG00000118922	KLF12	ENSG00000206596	RNU1-27P	ENSG00000169495	HTRA4
ENSG00000183889	PKD1P1	ENSG00000153310	FAM49B	ENSG00000163534	FCRL1
ENSG00000233024	AC126755.2	ENSG00000226608	FTLP3	ENSG00000108823	SGCA
ENSG00000142089	IFITM3	ENSG00000183426	NPIPA1	ENSG00000092929	UNC13D
ENSG00000196993	NPIPB9	ENSG00000110455	ACCS	ENSG00000198833	UBE2J1
ENSG00000145868	FBXO38	ENSG00000116489	CAPZA1	ENSG00000101187	SLCO4A1
ENSG00000106341	PPP1R17	ENSG00000173915	ATP5MD	ENSG00000213918	DNASE1
ENSG00000160111	CPAMD8	ENSG00000132963	POMP	ENSG00000110719	TCIRG1
ENSG00000165731	RET	ENSG00000172673	THEMIS	ENSG00000198829	SUCNR1

ENSG00000198821	CD247	ENSG00000164342	TLR3	ENSG00000114738	MAPKAPK3
ENSG00000105887	MTPN	ENSG00000188042	ARL4C	ENSG00000174177	CTU2
ENSG00000188158	NHS	ENSG00000101868	POLA1	ENSG00000100100	PIK3IP1
ENSG00000254893	AC113404.3	ENSG00000106948	AKNA	ENSG00000189014	SHLD2P3
ENSG00000077585	GPR137B	ENSG00000104852	SNRNP70	ENSG00000204160	ZDHHC18
ENSG00000162129	CLPB	ENSG00000164111	ANXA5	ENSG00000123728	RAP2C
ENSG00000007312	CD79B	ENSG00000163743	RCHY1	ENSG00000141337	ARSG
ENSG00000058056	USP13	ENSG00000211829	TRDC	ENSG00000131018	SYNE1
ENSG00000115568	ZNF142	ENSG00000087086	FTL	ENSG00000197057	DTHD1
ENSG00000078401	EDN1	ENSG00000142166	IFNAR1	ENSG00000258820	AF111167.1
ENSG00000104972	LILRB1	ENSG00000026103	FAS	ENSG00000149527	PLCH2
ENSG00000181143	MUC16	ENSG00000180739	S1PR5	ENSG00000074966	TXK
ENSG00000162645	GBP2	ENSG00000119922	IFIT2	ENSG00000144655	CSRNP1
ENSG00000177191	B3GNT8	ENSG00000168016	TRANK1	ENSG00000156273	BACH1
ENSG00000025039	RRAGD	ENSG00000206588	RNU1-28P	ENSG00000170365	SMAD1
ENSG00000272391	POM121C	ENSG00000198792	TMEM184B	ENSG00000139211	AMIGO2
ENSG00000123131	PRDX4	ENSG00000163378	EOGT	ENSG00000163577	EIF5A2
ENSG00000173068	BNC2	ENSG00000119698	PPP4R4	ENSG00000171916	LGALS9C
ENSG00000118680	MYL12B	ENSG00000054392	HHAT	ENSG00000183793	NPIPA5
ENSG00000065485	PDIA5	ENSG00000156475	PPP2R2B	ENSG00000205542	TMSB4X
ENSG00000108773	KAT2A	ENSG00000200169	RNU5D-1	ENSG00000174405	LIG4
ENSG00000164674	SYTL3	ENSG00000062598	ELMO2	ENSG00000160190	SLC37A1
ENSG00000008869	HEATR5B	ENSG00000256745	AP002784.2	ENSG00000186818	LILRB4
ENSG00000135363	LMO2	ENSG00000160014	CALM3	ENSG00000204472	AIF1
ENSG00000223609	HBD	ENSG00000133105	RXFP2	ENSG00000270022	Z93241.1
ENSG00000105246	EBI3	ENSG00000174231	PRPF8	ENSG00000164099	PRSS12
ENSG00000166394	CYB5R2	ENSG00000090565	RAB11FIP3	ENSG00000153107	ANAPC1
ENSG00000139182	CLSTN3	ENSG00000117228	GBP1	ENSG00000161057	PSMC2
ENSG00000124406	ATP8A1	ENSG00000143126	CELSR2	ENSG00000108582	CPD
ENSG00000127314	RAP1B	ENSG00000179639	FCER1A	ENSG00000111237	VPS29
ENSG00000124466	LYPD3	ENSG00000162413	KLHL21	ENSG00000176871	WSB2
ENSG00000125740	FOSB	ENSG00000207205	RNVU1-15	ENSG00000109390	NDUFC1
ENSG00000171163	ZNF692	ENSG00000174428	GTF2IRD2B	ENSG00000128944	KNSTRN
ENSG00000143776	CDC42BPA	ENSG00000205810	KLRC3	ENSG00000091106	NLRC4
ENSG00000198910	L1CAM	ENSG00000178175	ZNF366	ENSG00000215845	TSTD1
ENSG00000080298	RFX3	ENSG00000214194	SMIM30	ENSG00000009694	TENM1
ENSG00000152454	ZNF256	ENSG00000152672	CLEC4F	ENSG00000135900	MRPL44
ENSG00000174775	HRAS	ENSG00000211751	TRBC1	ENSG00000187653	TMSB4XP8
ENSG00000154265	ABCA5	ENSG00000140718	FTO	ENSG00000114446	IFT57
ENSG00000170385	SLC30A1	ENSG00000135838	NPL	ENSG00000196586	MYO6
ENSG00000182557	SPNS3	ENSG00000168229	PTGDR	ENSG00000181722	ZBTB20

ENSG00000147155	EBP	ENSG00000125898	FAM110A	ENSG00000276966	HIST1H4E
ENSG00000111348	ARHGDIB	ENSG00000148484	RSU1	ENSG00000246705	H2AFJ
ENSG00000115523	GNLY	ENSG00000102794	ACOD1	ENSG00000265666	RARA-AS1
ENSG00000172757	CFL1	ENSG00000182866	LCK	ENSG00000069702	TGFBR3
ENSG00000178921	PFAS	ENSG00000104687	GSR	ENSG00000065911	MTHFD2
ENSG00000115652	UXS1	ENSG00000119013	NDUFB3	ENSG00000143353	LYPLAL1
ENSG00000167315	ACAA2	ENSG00000198851	CD3E	ENSG00000108515	ENO3
ENSG00000166398	KIAA0355	ENSG00000279602	AC109326.1	ENSG00000160654	CD3G
ENSG00000143771	CNIH4	ENSG00000100985	MMP9	ENSG00000100450	GZMH
ENSG00000136111	TBC1D4	ENSG00000133247	KMT5C	ENSG00000077463	SIRT6
ENSG00000138002	IFT172	ENSG00000254852	NPIPA2	ENSG00000157388	CACNA1D
ENSG00000213281	NRAS	ENSG00000100266	PACSIN2	ENSG00000162704	ARPC5
ENSG00000197948	FCHSD1	ENSG00000131165	CHMP1A	ENSG00000168884	TNIP2
ENSG00000189350	TOGARAM2	ENSG00000128340	RAC2		
ENSG00000152270	PDE3B				

Supplementary Table 3. List of significant DEGs in the Adult acute malaria cohort (FDR<0.05)

Ensemble gene ID	Gene symbol	Ensemble gene ID	Gene symbol	Ensemble gene ID	Gene symbol
ENSG00000154258	ABCA9	ENSG00000166501	PRKCB	ENSG00000113749	HRH2
ENSG00000133067	LGR6	ENSG00000166387	PPFIBP2	ENSG00000196839	ADA
ENSG00000102962	CCL22	ENSG00000127507	ADGRE2	ENSG00000145416	MARCH1
ENSG00000164483	SAMD3	ENSG00000116337	AMPD2	ENSG00000139194	RBP5
ENSG00000114204	SERPINI2	ENSG00000172932	ANKRD13D	ENSG00000126266	FFAR1
ENSG00000278528	AC145285.7	ENSG00000135596	MICAL1	ENSG00000166446	CDYL2
ENSG00000120337	TNFSF18	ENSG00000179241	LDLRAD3	ENSG00000126458	RRAS
ENSG00000073861	TBX21	ENSG00000147155	EBP	ENSG00000141401	IMPA2
ENSG00000164929	BAALC	ENSG00000198055	GRK6	ENSG00000161955	TNFSF13
ENSG00000211751	TRBC1	ENSG00000163171	CDC42EP3	ENSG00000174837	ADGRE1
ENSG00000163739	CXCL1	ENSG00000102543	CDADC1	ENSG00000197461	PDGFA
ENSG00000278078	AC025279.2	ENSG00000141232	TOB1	ENSG00000172985	SH3RF3
ENSG00000261203	AC106782.3	ENSG00000185201	IFITM2	ENSG00000112303	VNN2
ENSG00000261419	AC145285.4	ENSG00000109103	UNC119	ENSG00000101187	SLCO4A1
ENSG00000069493	CLEC2D	ENSG00000106348	IMPDH1	ENSG00000130475	FCHO1
ENSG00000200169	RNU5D-1	ENSG00000077585	GPR137B	ENSG00000174945	AMZ1
ENSG00000155659	VSIG4	ENSG00000110046	ATG2A	ENSG00000093134	VNN3
ENSG00000118200	CAMSAP2	ENSG00000174791	RIN1	ENSG00000213889	PPM1N
ENSG00000140287	HDC	ENSG00000159496	RGL4	ENSG00000153208	MERTK
ENSG00000262202	AC007952.4	ENSG00000165752	STK32C	ENSG00000162073	PAQR4
ENSG00000196569	LAMA2	ENSG00000145476	CYP4V2	ENSG00000007968	E2F2
ENSG00000172183	ISG20	ENSG00000089060	SLC8B1	ENSG00000077522	ACTN2
ENSG00000224769	MUC20P1	ENSG00000122122	SASH3	ENSG00000172594	SMPDL3A

ENSG00000257506	AC133555.1	ENSG00000135363	LMO2	ENSG00000142512	SIGLEC10
ENSG00000166689	PLEKHA7	ENSG00000197321	SVIL	ENSG00000175489	LRRC25
ENSG00000211942	IGHV3-13	ENSG00000028137	TNFRSF1B	ENSG00000169495	HTRA4
ENSG00000157404	KIT	ENSG00000095370	SH2D3C	ENSG00000105173	CCNE1
ENSG00000118513	MYB	ENSG00000139182	CLSTN3	ENSG00000165548	TMEM63C
ENSG00000198795	ZNF521	ENSG00000018280	SLC11A1	ENSG00000105048	TNNT1
ENSG00000133316	WDR74	ENSG00000095015	MAP3K1	ENSG00000173372	C1QA
ENSG00000279602	AC109326.1	ENSG00000182957	SPATA13	ENSG00000174705	SH3PXD2B
ENSG00000173193	PARP14	ENSG00000032444	PNPLA6	ENSG00000234424	AL353743.4
ENSG00000248323	LUCAT1	ENSG00000167552	TUBA1A	ENSG00000182885	ADGRG3
ENSG00000186017	ZNF566	ENSG00000065154	OAT	ENSG00000276758	AC245884.12
ENSG00000196139	AKR1C3	ENSG00000160883	HK3	ENSG00000002933	TMEM176A
ENSG00000166881	NEMP1	ENSG00000088992	TESC	ENSG00000205213	LGR4
ENSG00000203761	MSTO2P	ENSG00000167083	GNGT2	ENSG00000244405	ETV5
ENSG00000184988	TMEM106A	ENSG00000144579	CTDSP1	ENSG00000134061	CD180
ENSG00000125459	MSTO1	ENSG00000134256	CD101	ENSG00000108950	FAM20A
ENSG00000013374	NUB1	ENSG00000106789	CORO2A	ENSG00000198719	DLL1
ENSG00000179344	HLA-DQB1	ENSG00000198879	SFMBT2	ENSG00000007312	CD79B
ENSG00000152784	PRDM8	ENSG00000146094	DOK3	ENSG00000106565	TMEM176B
ENSG00000124813	RUNX2	ENSG00000120949	TNFRSF8	ENSG00000110079	MS4A4A
ENSG00000124575	HIST1H1D	ENSG00000105639	JAK3	ENSG00000173369	C1QB
ENSG00000152518	ZFP36L2	ENSG00000111144	LTA4H	ENSG00000261701	HPR
ENSG00000169131	ZNF354A	ENSG00000072274	TFRC	ENSG00000169715	MT1E
ENSG00000142961	MOB3C	ENSG00000186074	CD300LF	ENSG00000135424	ITGA7
ENSG00000092929	UNC13D	ENSG00000105612	DNASE2	ENSG00000257017	HP
ENSG00000160796	NBEAL2	ENSG00000100373	UPK3A	ENSG00000125144	MT1G
ENSG00000106868	SUSD1	ENSG00000159640	ACE	ENSG00000170439	METTL7B
